# Formation and characterization of BMP2/GDF5 and BMP4/GDF5 heterodimers

**DOI:** 10.1186/s12915-023-01522-4

**Published:** 2023-02-01

**Authors:** Gregory R. Gipson, Kristof Nolan, Chandramohan Kattamuri, Alan P. Kenny, Zachary Agricola, Nicole A. Edwards, Joseph Zinski, Magdalena Czepnik, Mary C. Mullins, Aaron M. Zorn, Thomas B. Thompson

**Affiliations:** 1 Department of Molecular & Cellular Biosciences, University of Cincinnati College of Medicine, Cincinnati, OH USA; 2 Department of Biochemistry and Molecular Biophysics, University of Chicago, Chicago, IL USA; 3 Perinatal Institute, Divisions of Developmental Biology and Neonatology & Pulmonary Biology, Cincinnati Children’s Hospital Medical Center, Cincinnati, OH USA; 4 Department of Cell and Developmental Biology, University of Pennsylvania Perelman School of Medicine, Philadelphia, PA USA

**Keywords:** BMP, Growth factor, Heterodimer, Cell signaling

## Abstract

**Background:**

Proteins of the TGFβ family, which are largely studied as homodimers, are also known to form heterodimers with biological activity distinct from their component homodimers. For instance, heterodimers of bone morphogenetic proteins, including BMP2/BMP7, BMP2/BMP6, and BMP9/BMP10, among others, have illustrated the importance of these heterodimeric proteins within the context of TGFβ signaling.

**Results:**

In this study, we have determined that mature GDF5 can be combined with mature BMP2 or BMP4 to form BMP2/GDF5 and BMP4/GDF5 heterodimer. Intriguingly, this combination of a BMP2 or BMP4 monomer, which exhibit high affinity to heparan sulfate characteristic to the BMP class, with a GDF5 monomer with low heparan sulfate affinity produces a heterodimer with an intermediate affinity. Using heparin affinity chromatography to purify the heterodimeric proteins, we then determined that both the BMP2/GDF5 and BMP4/GDF5 heterodimers consistently signaled potently across an array of cellular and in vivo systems, while the activities of their homodimeric counterparts were more context dependent. These differences were likely driven by an increase in the combined affinities for the type 1 receptors, Alk3 and Alk6. Furthermore, the X-ray crystal structure of BMP2/GDF5 heterodimer was determined, highlighting the formation of two asymmetric type 1 receptor binding sites that are both unique relative to the homodimers.

**Conclusions:**

Ultimately, this method of heterodimer production yielded a signaling molecule with unique properties relative to the homodimeric ligands, including high affinity to multiple type 1 and moderate heparan binding affinity.

**Supplementary Information:**

The online version contains supplementary material available at 10.1186/s12915-023-01522-4.

## Background

The transforming growth factor β (TGFβ) superfamily represents one of the largest and most fundamental signaling families in human biology, comprised of over 30 unique signaling ligands [1–5]. This family can be subdivided into three main subgroups: the bone morphogenetic protein (BMP) class, the Activin class, and the TGFβ class [4–6]. While each maintains a similar overall structure and signaling mechanism, BMPs are unique both in terms of specific functional biochemistry and the biological processes they regulate [6, 7]. BMPs regulate numerous developmental processes such as bone and cartilage formation, organ development, and overall developmental patterning [2, 3, 8] In adult biology, BMPs are equally vital, regulating a host of processes involved in wound healing and cell homeostasis [2, 3, 8]. When BMP signaling is compromised, a number of disease state pathologies can occur, including renal nephropathy and fibrosis, pulmonary arterial hypertension, cardiovascular disease and atherosclerosis, osteoporosis, developmental cartilage disorders, and numerous types of cancer [8–18]. Therapeutically, different forms of recombinant BMP proteins are used in clinical settings to treat traumatic bone injuries [2, 8, 17, 18].

Like other TGFβ proteins, BMPs are produced as single-chain polypeptides consisting of a larger pro-domain (30–40 kDa) and a smaller (12–15 kDa) mature signaling domain separated by one or more proteolytic cleavage sites [4, 6, 7, 19–21]. Prodomain interactions cause the mature domains to covalently dimerize before being secreted from the cell where they are proteolytically cleaved allowing the mature signaling domains bind receptors [22, 23]. Signaling occurs when the mature domain, structurally reminiscent of two clasped hands complete with four β-strand “fingers” and a “wrist” helix for each monomer, combines two type 1 and two type 2 serine-threonine kinase receptors on the cell surface leading to intracellular cross-phosphorylation of the type 1 receptors and subsequent phosphorylation of specific SMAD transcription factors [4–7, 24].

While all BMPs function along this general mechanism, a number of different factors are key to the modulation of signaling for each distinct ligand. Different BMPs have different preferences for both the high-affinity type 1 and lower-affinity type 2 receptors [24]. For example, BMP2 can signal through the type 1 receptors Alk3 and Alk6 but maintains a higher affinity for Alk3 [24, 25]. Growth and Differentiation Factor 5 (GDF5), on the other hand, strongly prefers signaling through Alk6 as compared to Alk3, leading to differences in signaling outcomes [26]. BMPs are also differentially targeted by a host of secreted extracellular protein antagonists, such as Noggin, Chordin, and members of the DAN family [4, 27–30]. For instance, Gremlin-2 and Noggin show much higher affinity for BMP2 over GDF5 although signaling from either ligand is inhibited [27, 28]. Lastly, BMPs can interact differently with the extracellular environment, where proteins like BMP2 or BMP7 possess high affinity for heparin and heparan sulfate proteoglycans (HS), while others like BMP9 or GDF5 do not [31–34]. As a result, BMPs with lower HS affinity are much more likely to diffuse to neighboring cells and/or circulate widely while those with higher affinity interact with the extracellular matrix and are more restricted in their diffusion.

**Fig. 1:**
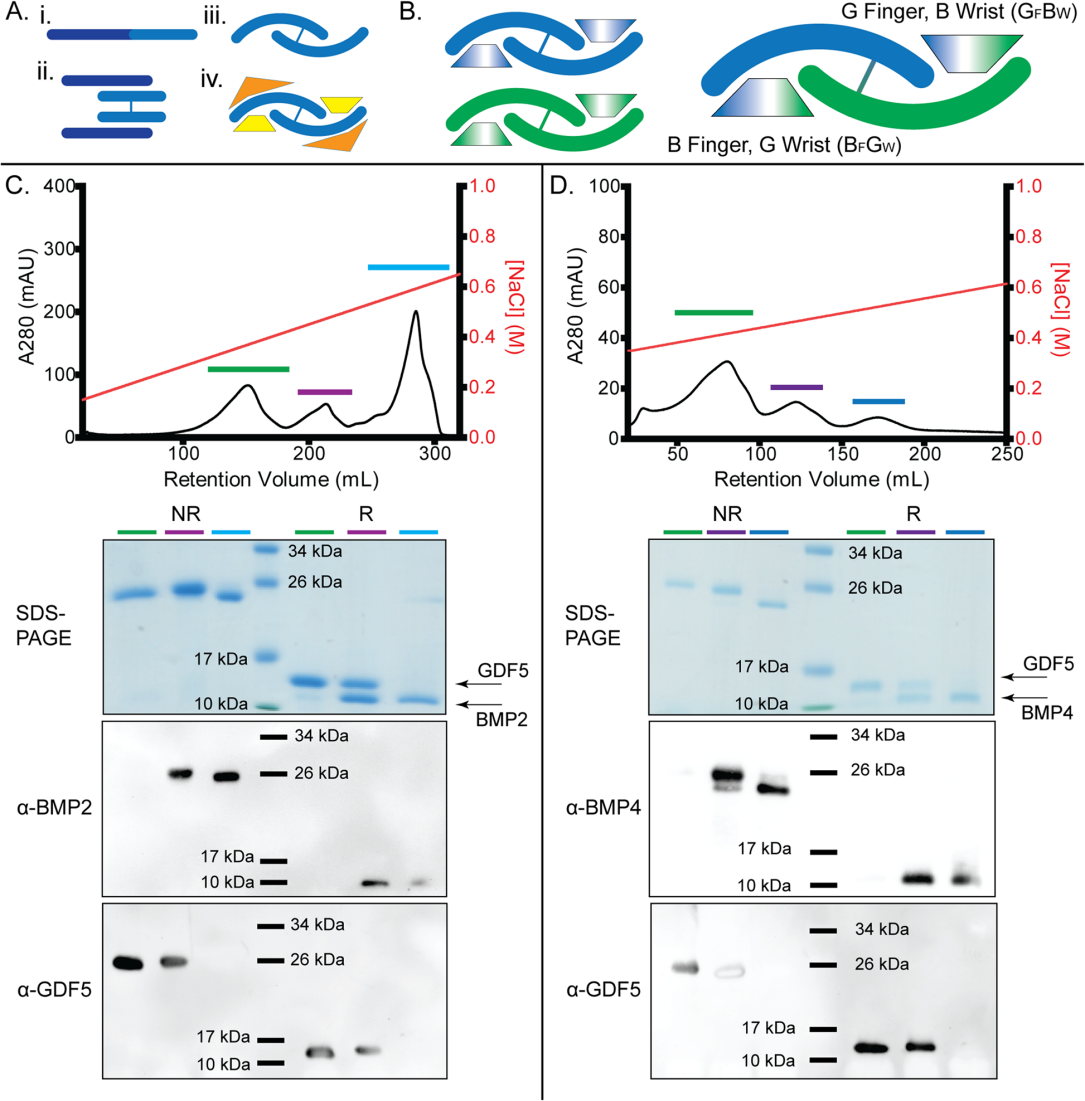
Schematic of native BMP production and heterodimer fabrication. **A** Schematic of the production and function of a BMP growth factor. (i) BMPs are produced as a single polypeptide chain consisting of a larger prodomain (30–40 kDa, dark blue) and a smaller (12–14 kDA, light blue) mature domain. (ii) Prodomain interactions bring the mature domains together and induce dimerization, which is stabilized by an inter-chain disulfide bond. (iii) representation of the mature ligand and shown in iv) bound to two high-affinity type 1 receptors (yellow) and two low-affinity type 2 receptors (orange). **B** Schematic of differences in type 1 interfaces of homo- and hetero-dimers. The heterodimer (BMP chain in blue and GDF5 chain in green) consists of two unique binding sites, one formed by the fingers of BMP and the wrist of GDF5 and one formed by the fingers of GDF5 and wrist of BMP, as annotated. **C** Heparin affinity chromatography is used to separate refolded homodimeric GDF5 (green) and homodimeric BMP2 (blue) from BMP2/GDF5 heterodimer (purple) based on differential heparin binding affinity. Presence of pure homodimeric and heterodimeric proteins confirmed by SDS-PAGE and western blot analysis, under both reducing and non-reducing conditions. **D** Heparin affinity chromatography is used to separate refolded homodimeric GDF5 (green) and homodimeric BMP4 (blue) from BMP4/GDF5 heterodimer (purple) based on differential heparin binding affinity. Presence of pure homodimeric and heterodimeric proteins confirmed by SDS-PAGE and western blot analysis, under both reducing and non-reducing conditions. Protein ladder shown at 10 kDa, 17 kDa, 26 kDa, and 34 kDa

While most previous research has focused on BMP ligands as homodimers, recent work has extended to a small number of specific BMP heterodimers. These heterodimers produce a unique signaling platform with biological activities distinct from their homodimeric counterparts [35–39]. One of the major functional differences of the heterodimers occurs with type 1 receptor binding/signaling. Structural studies have highlighted that type 1 receptor binding occurs at the dimer interface of these ligands [40, 41]. Since this is a composite interface from both monomers, a heterodimer will presumably contain two unique type 1 receptor binding sites, distinct from both each other within the heterodimer and with their corresponding homodimers. While this is shown schematically in Fig. 1A, B, the extent and details of such a dimeric interface have yet to be structurally elucidated.

Evidence for the biological significance of heterodimers have been shown by several groups over the past several years. For example, the BMP2/BMP7 heterodimer has been shown to serve as the essential growth factor in developing zebrafish, with homodimeric BMP2 and BMP7 unable to recapitulate this lost activity [36]. BMP7 seems to function primarily as a heterodimer with either BMP2 or BMP4 in mammalian embryogenesis [37]. BMP2/BMP6 has been shown to be a more potent activator of SMAD 1/5/9 signaling than either BMP2 or BMP6 homodimers, and to potentially play a novel role in iron regulation [36, 42]. BMP9/BMP10 heterodimer has been shown to be the principal BMP ligand involved in signaling in the serum [33]. Furthermore, heterodimeric GDF9/BMP15, also known as cumulin, has the ability to activate both SMAD 1/5/9 and SMAD 2/3, acting as both a BMP and an activin signaling molecule [43, 44].

Research on heterodimers is challenging since homodimers are synthesized concurrently with heterodimers often making it difficult to discern the functions of heterodimers from homodimers, leaving many potential heterodimers completely unclassified or even observed. Recombinant techniques are useful in generating purified heterodimers which can be used to support their characterization; however, the number of heterodimers available is limited. Accordingly, previous research into BMP heterodimers has by necessity focused on these few listed examples. Because of this, little research has focused on whether the subclade of BMPs containing GDF5, GDF6, and GDF7 can form heterodimers. One early study identified the ability of GDF5 to heterodimerize with BMP2, BMP3, and BMP7 where immunoprecipitation experiments were performed on cells overexpressing BMP ligands, suggesting the existence of GDF/BMP heterodimers [45]. However, follow-up studies are absent, and no work has been done to characterize GDF/BMP heterodimers.

With this in mind, we sought to investigate the BMP2/GDF5 heterodimer and explore its functionality in comparison to its homodimer counterparts. As such, we have produced a BMP2/GDF5 heterodimer using oxidative refolding of bacterially produced mature domains and demonstrated its unique biological activity compared to BMP2 and GDF5 using in vitro reporter assays, direct binding experiments, and in vivo developmental assays. Additionally, we validated our method by also producing the BMP4/GDF5 heterodimer and demonstrated that it also forms a more robust signaling platform than either BMP4 or GDF5 alone. Lastly, we solved the crystal structure of the BMP2/GDF5 heterodimer, giving molecular insight into how heterodimers form asymmetrical type I binding pockets. Taken together, our structure and functional data provide a platform for describing the different signaling aptitude of the BMP2/GDF5 and BMP4/GDF5 heterodimers in comparison to the BMP2, BMP4, and GDF5 homodimers.

## Results

### Production and validation of GDF5/BMP2 and GDF5/BMP4 heterodimers

While the previous study by Thomas et al. indicated that GDF5 could form heterodimers with other BMPs, no further published work exists exploring the possibility of heterodimers made with GDF5 [45]. Since both BMP2 and GDF5 homodimers can be produced and purified from oxidative refolding from bacterial inclusion bodies, we hypothesized that a modified oxidative refolding protocol could be implemented to form a BMP2/GDF5 heterodimer [46, 47]. BMP2 and GDF5 inclusion bodies were purified and solubilized and then mixed in a 1:1 molar ratio prior to oxidative refolding. The mixture of BMP2 and GDF5 was then allowed to refold for 5 days. Following refolding, BMP2 and GDF5 homodimers are typically separated from improperly folded species by heparin affinity chromatography where BMP2 dimers are eluted with high salt while GDF5 binds weaker and is eluted with lower salt concentrations [31, 34]. We hypothesized that a heterodimer might exhibit an intermediate affinity for heparin. Accordingly, we applied the refolding mixture of BMP2/GDF5 to a heparin column and three separate peaks were identified upon elution (Fig. 1C). Analysis by SDS-PAGE and Western blots, under reducing and nonreducing conditions, indicated the first peak contained GDF5 homodimer and the last peak contained BMP2 homodimer. The intermediate peak consisted of a single dimer species containing both BMP2 and GDF5 chains (Fig. 1C). To rule out that the intermediate peak consisted of GDF5 and BMP2 homodimers instead of a BMP2/GDF5 heterodimer, we analyzed the sample by mass spectrometry (Additional file 1: Fig. S1). The results show a single species with a mass consistent with the expected mass of a heterodimer. In addition, to validate our approach of heterodimer formation, we repeated the process with BMP4 and GDF5. Similarly, three peaks were identified from the heparin elution profile with the middle peak consisting of the BMP4/GDF5 heterodimer (Fig. 1D, Additional file 1: Fig. S1). These results show that both BMP2/GDF5 and BMP4/GDF5 heterodimers can be produced thorough oxidative refolding and isolated to homogeneity for further analysis.

### Comparison between heterodimer and homodimer signaling in vitro

**Fig. 2:**
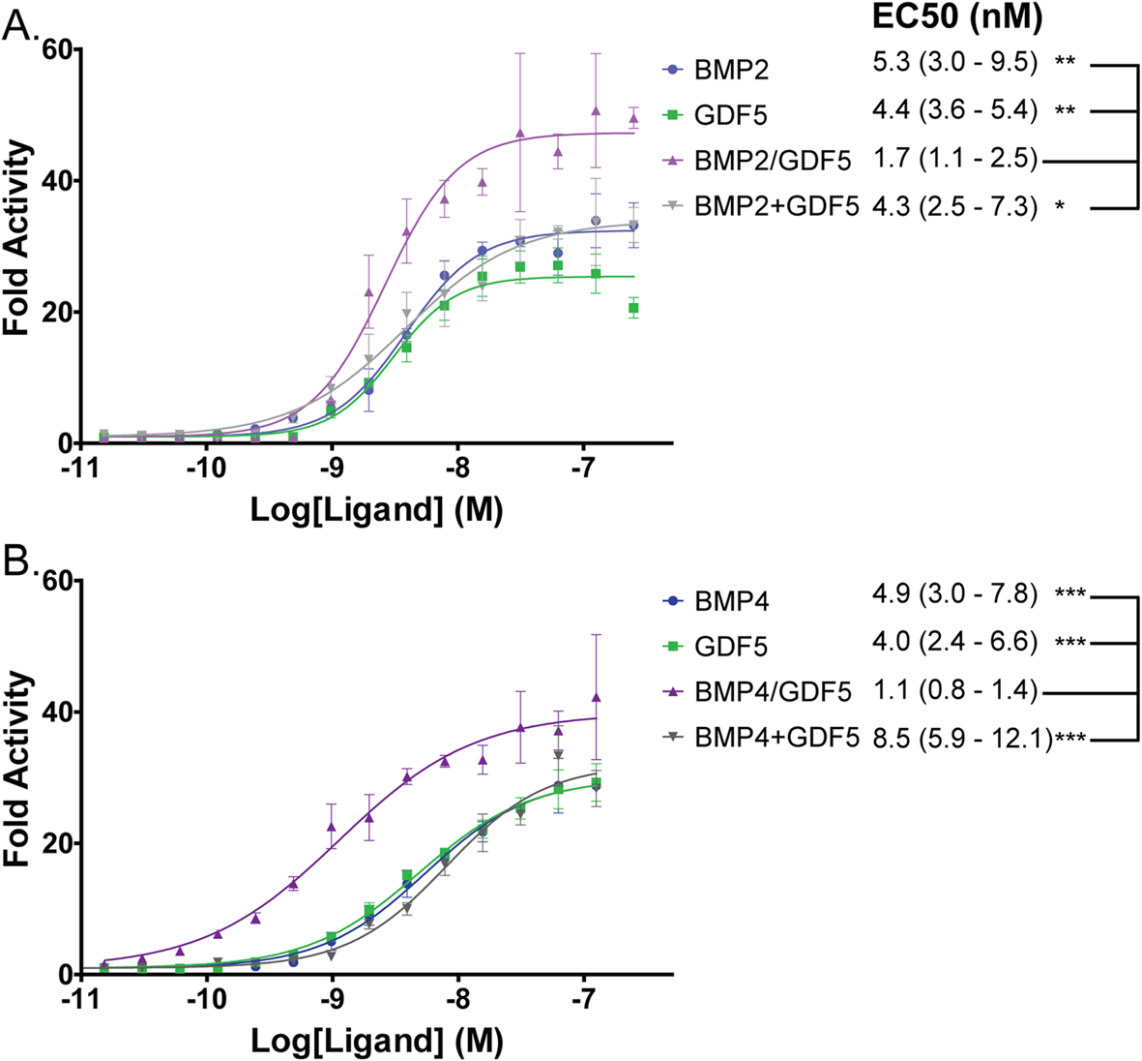
Heterodimers are more potent than homodimers in vitro. **A** Luciferase Reporter Assay used to compare function of BMP2/GDF5 heterodimer (purple) to BMP2 homodimer (blue), GDF5 homodimer (green), or a combination of BMP2 and GDF5 homodimers (gray). The gray curve represents signal from an equal combination of BMP and GDF5 where the sum is represented on the x-axis. **B** Luciferase Reporter Assay used to compare function of BMP4/GDF5 heterodimer (purple) to BMP4 homodimer (blue), GDF5 homodimer (green), or a combination of BMP2 and GDF5 homodimers (gray). Representative curves shown, with error bars representing standard deviation. Data normalized to untreated control and analyzed using GraphPad Prism using non-linear regression with variable slope and least squares fit to determine EC_50_. Data tables display an average of *N*=3 experiments, with 95% confidence range reported. All datasets were compared to heterodimer to determine significance using AICc to compare average curves. * *P*<0.05. ** *P*<0.01. *** *P*<0.001

Previous research performed on other BMP heterodimers (specifically BMP2/7 and BMP2/6) found that heterodimers tend to be more potent activators of BMP signaling than their homodimer counterparts [35–37]. To determine whether this was true for the BMP2/GDF5 and BMP4/GDF5 heterodimers we tested signaling activity using the BRITER osteoblast cell line. The BRITER cell line has been engineered to express luciferase in response to BMP signaling via SMAD 1/5/9 [48]. In this assay system, both the BMP2/GDF5 and BMP4/GDF5 heterodimers are potent signaling molecules with EC_50_ values of 1.7 and 1.1 nM, respectively (Fig. 2). In comparison to the BMP2, BMP4, or GDF5 homodimers, the heterodimers are significantly more potent with EC_50_ values approximately 3–5-fold lower than all homodimers (Fig. 2). Additionally, the BMP2/GDF5 and BMP4/GDF5 heterodimers signal more potently than cells incubated with molar equivalents of homodimeric BMP2 plus GDF5 or BMP4 plus GDF5 added in combination (Fig. 2). Furthermore, at higher ligand concentrations the heterodimers exhibited greater maximum signal than their respective homodimers.

### Comparison between heterodimer and homodimer signaling in vivo

**Fig. 3:**
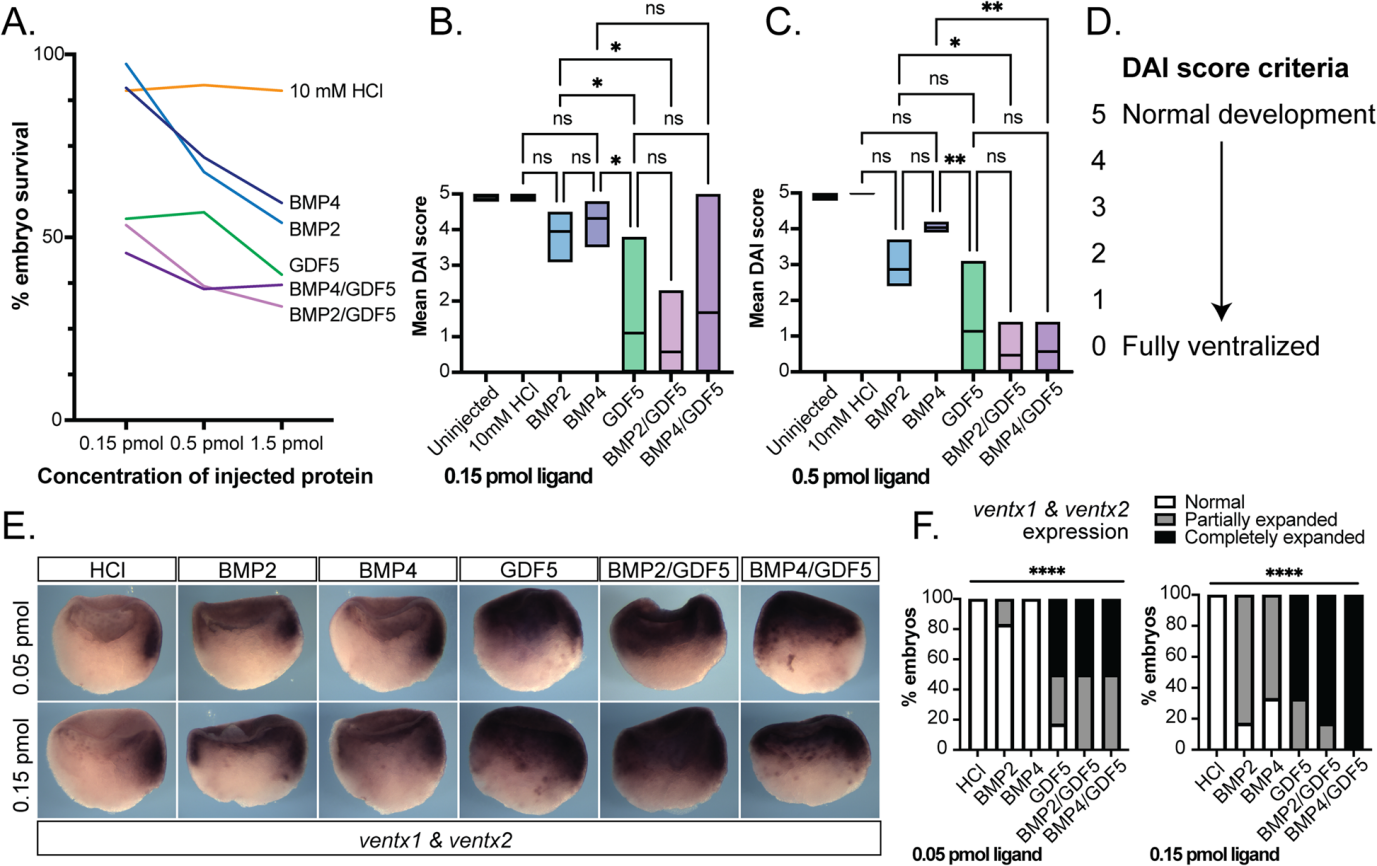
Heterodimers signal more potently than BMP2 and BMP4 homodimers in a *Xenopus* development assay. **A** Dose-response experiment determined the minimal effective dose of homodimers/heterodimers for developmental assays. Based on embryo viability, 0.5 pmol was the highest dose used for subsequent assays. **B**, **C** *Xenopus* axial development assay scored using Dorso-Anterior Index [45]. Stage 9 *Xenopus laevis* blastula were injected with 0.15 pmol in 13 nL (**B**) or 0.5 pmol in 40 nL doses (**C**) of BMP homodimeric or heterodimeric proteins in 10 mM HCl, or a vehicle control. Embryos developed until stage NF37 (tadpole stage) and were then scored using the (**D**) Dorso-Anterior Index for signs of aberrant ventralization, on a scale of 5 (normal development) to 0 (fully ventralized, no visible somites) [49]. *p<0.05, **p<0.01 by 1W-ANOVA, *n*=10–15 embryos, *N*=3–4 independent experiments. **E** *N*=20 stage NF9 *Xenopus laveis* blastula microinjected 0.05 pmol in 4.3 nL or 0.15 pmol in 13 nL of BMP homodimeric or heterodimeric proteins or vehicle control were assayed at early gastrula stage NF10, by *in situ* hybridization for expression of direct BMP/SMAD1 response genes *ventx1* and *ventx2* (dark staining). Ventral side to the right. Representative images shown. **F** Gastrula embryos were scored for degree of *ventx1* and *ventx2* staining expression and categorized as normal, partially expanded, or completely expanded expression domains. ****p<0.0001 by chi-square test *n*=20 embryos, *N*=1 experiment

Having determined that the BMP/GDF heterodimers induce BMP signaling more potently than their component homodimers in a cell-based assay, it was important to determine whether these findings could be validated in an in vivo model more closely replicating a complete biological system. The role of BMP signaling in the patterning of the embryonic body axis in *Xenopus laevis* is extremely well characterized, and perturbations caused by aberrant signaling have been frequently used as an assay system to measure signaling activity in vivo [49, 50]. Accordingly, we used a *Xenopus* development assay to measure the activity of BMP homodimers versus heterodimers. Recombinant ligands or a vehicle control were microinjected injected into the blastocoel of *Xenopus* blastula and permitted to develop for ~46 h until they reached the tadpole stage, stage NF37, when they were scored for ventralization. First, a dose-response experiment was performed to determine the minimal effective dose eliciting a phenotype with minimal lethality; extremely ventralized embryos arrest at gastrulation and subsequently die (Fig. 3A). Embryos injected with 0.15 pmol and 0.5 pmol protein were blindly scored using the dorso-anterior index (DAI), which can measure the degree of aberrant embryonic ventralization [49]. In this scoring system, a score of 5 indicates normal development and a score of 0 indicates the most severe degree of ventralization (Fig. 3D).

Gastrulation arrest and lethality were more frequent in embryos injected with heterodimers as compared to BMP2 and BMP4, and slightly more than GDF5 homodimer (Fig. 3A). Consistent with previous results, the injection of recombinant BMP2 or BMP4 homodimers resulted in increased ventralization in a concentration-dependent manner [51]. Interestingly, in this assay system, GDF5 homodimers caused more severe ventralization than either BMP2 or BMP4 (Fig. 3B, C). This is in contrast to the in vitro system, where BMP2 was the most potent of the homodimers (Fig. 2). Similar to GDF5, injection of either the BMP2/GDF5 or BMP4/GDF5 heterodimers produced a dose-dependent increase in ventralization compared to the BMP2 and BMP4 homodimers at all tested concentrations (Fig. 3B, C).

In the *Xenopus* embryo, BMP/SMAD signaling is known to directly activate the expression of *ventx1* and *ventx2* genes that drive ventral cell fates in the gastrula mesendoderm [52, 53]. Therefor we assayed a subset of the *Xenopus* embryos at gastrulation, stage NF10, only a few hours post-injection, using whole mount in situ hybridization. In control embryos, *ventx1/2* expression is limited to the most ventral side of the developing gastrula (Fig. 3E). Injection of BMP2 and BMP4 resulted in an increasing expansion of *ventx1/2* expression in a dose-dependent manner. In contrast, injection of GDF5 or BMP/GDF5 heterodimers induced a dose-dependent expansion of the *ventx1/2* expression domain spreading across the top of the gastrula and into the dorsal side (Fig. 3E, F). This illustrates that in vivo both BMP2/GDF5 and BMP4/GDF5 are highly active compared to BMP2 and BMP4 homodimers (Fig. 3B).

We next extended our in vivo studies into a biological system that has been previously used to validate BMP heterodimer function and necessity. The dorsal-ventral axis of the zebrafish embryo is patterned by a gradient of BMP signaling established by BMP2/BMP7 heterodimers. BMP2 and BMP7 homodimers are unable to recapitulate this biological function and do not even signal at physiological levels, though either can induce BMP signaling when overexpressed [36, 54]. The BMP signaling gradient patterning the DV axis in zebrafish blastula has been previously quantified by visualizing the gradient of phosphorylated SMAD5 (pSMAD5) [55, 56]. The phosphorylation and nuclear accumulation of the SMAD5 protein are quantified by measuring the average nuclear fluorescence of the pSMAD5 antibody using confocal microscopy [57]. SMAD5 is an ideal readout of BMP signaling due to its rapid and robust phosphorylation and nuclear localization in response to BMP ligand, accumulating clearly within minutes of induction [58]. Accordingly, we sought to determine if, in a system where the BMP2/BMP7 heterodimer is the obligate signaling ligand, how the BMP2/GDF5 heterodimer signals compared to its component homodimers.

**Fig. 4:**
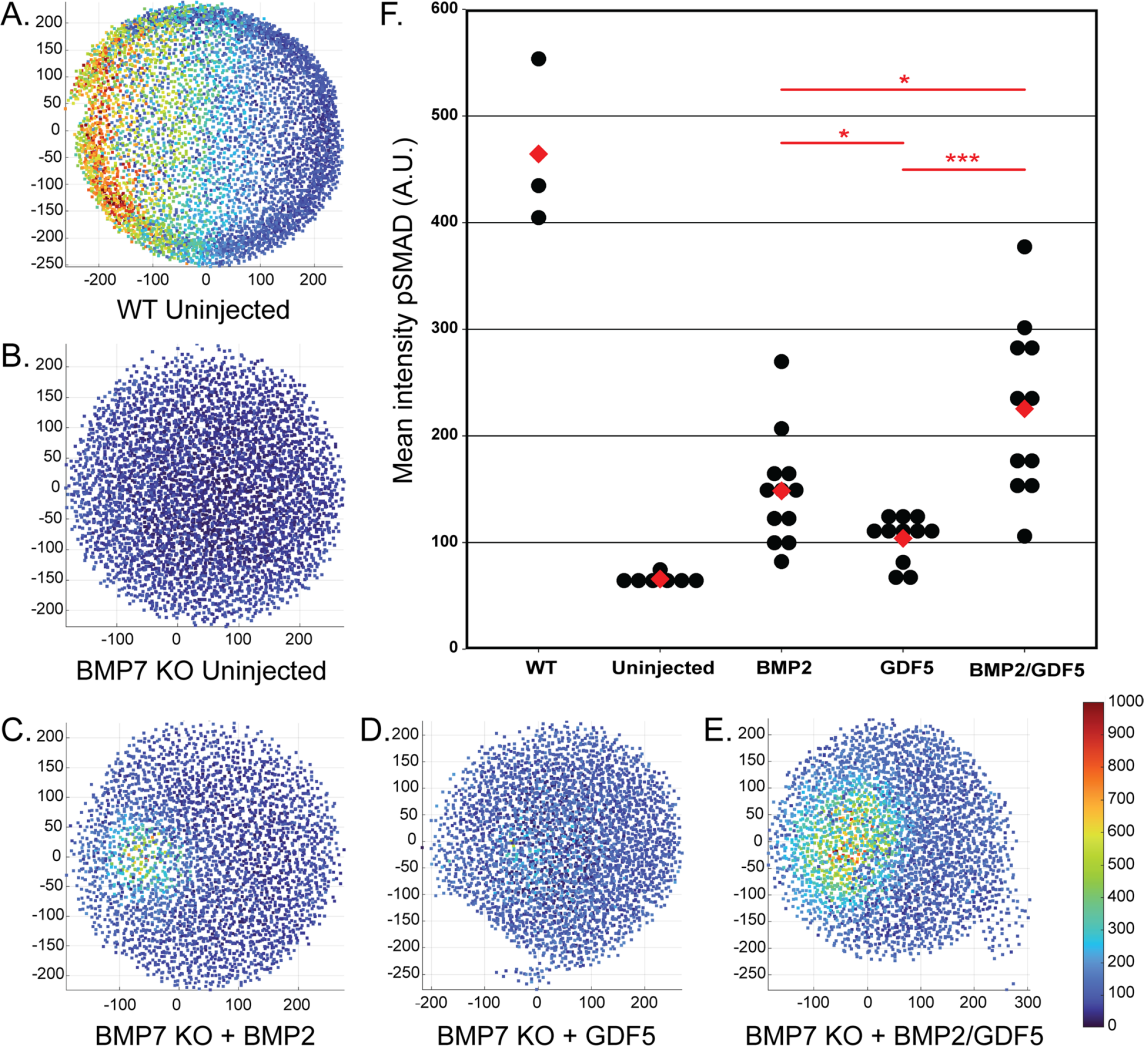
BMP2/GDF5 heterodimer signals more effectively than BMP2 or GDF5 homodimers in developing Zebrafish blastula. **A**–**E** Representative images of individual zebrafish embryos with quantitative fluorescence for nuclear phosphorylated SMAD5 (pSMAD5). Each dot depicts the average fluorescence of -pSMAD5 in a single nucleus, reds are brighter, and blues are dimmer. **A** WT embryo at 6 hpf. **B**–**E** *bmp7−/−* embryos at 3 hpf. Embryos were injected with 0.08 fmol protein and then fixed 30 min after injection. **F** Quantification of the nuclear pSMAD5 fluorescence in embryos was done by calculating the average intensity of the brightest 75% of nuclei minus the average intensity of the dimmest 25% (assumed background). Each black dot is a single embryo. Means are denoted by red diamonds. Experimental groups contained *n* = 3 (WT), 7 (uninjected), 12 (BMP2), 10 (GDF5), or 11 (BMP2/GDF5) embryos. *: *P*<0.05, ***: *P*<0.001 by T-test

To accomplish this, recombinant ligands (BMP2, GDF5, and BMP2/GDF5) were injected into zebrafish embryos, either WT or mutants deficient in BMP7 (*bmp7a*^*sb1aub*^) to negate endogenous BMP signaling, 3 hours post fertilization (hpf). After 30 min, the injected embryos, as well as uninjected WT and mutant controls, were quantified for nuclear pSMAD5 via immunofluorescence (Fig. 4). As expected, there was robust signaling in uninjected WT embryos (Fig. 4A) but none in uninjected BMP7 mutant embryos (Fig. 4B). Homodimeric BMP2 protein was injected at an experimentally determined dose of 0.08 fmol to induce a mild pSMAD5 response (Fig. 4C), midway between the extremes of the control embryos. Injection of homodimeric GDF5 at this dose induced a less pronounced pSMAD5 response compared to BMP2 (Fig. 4D). In contrast, injection of the BMP2/GDF5 heterodimer induced greater pSMAD5 response compared to either homodimer injected alone (Fig. 4E, F). These results suggest that the BMP2/GDF5 heterodimer can induce pSMAD5 signaling more strongly than either homodimer in this model system.

### Comparison of homodimer and heterodimer receptor binding

**Fig. 5:**
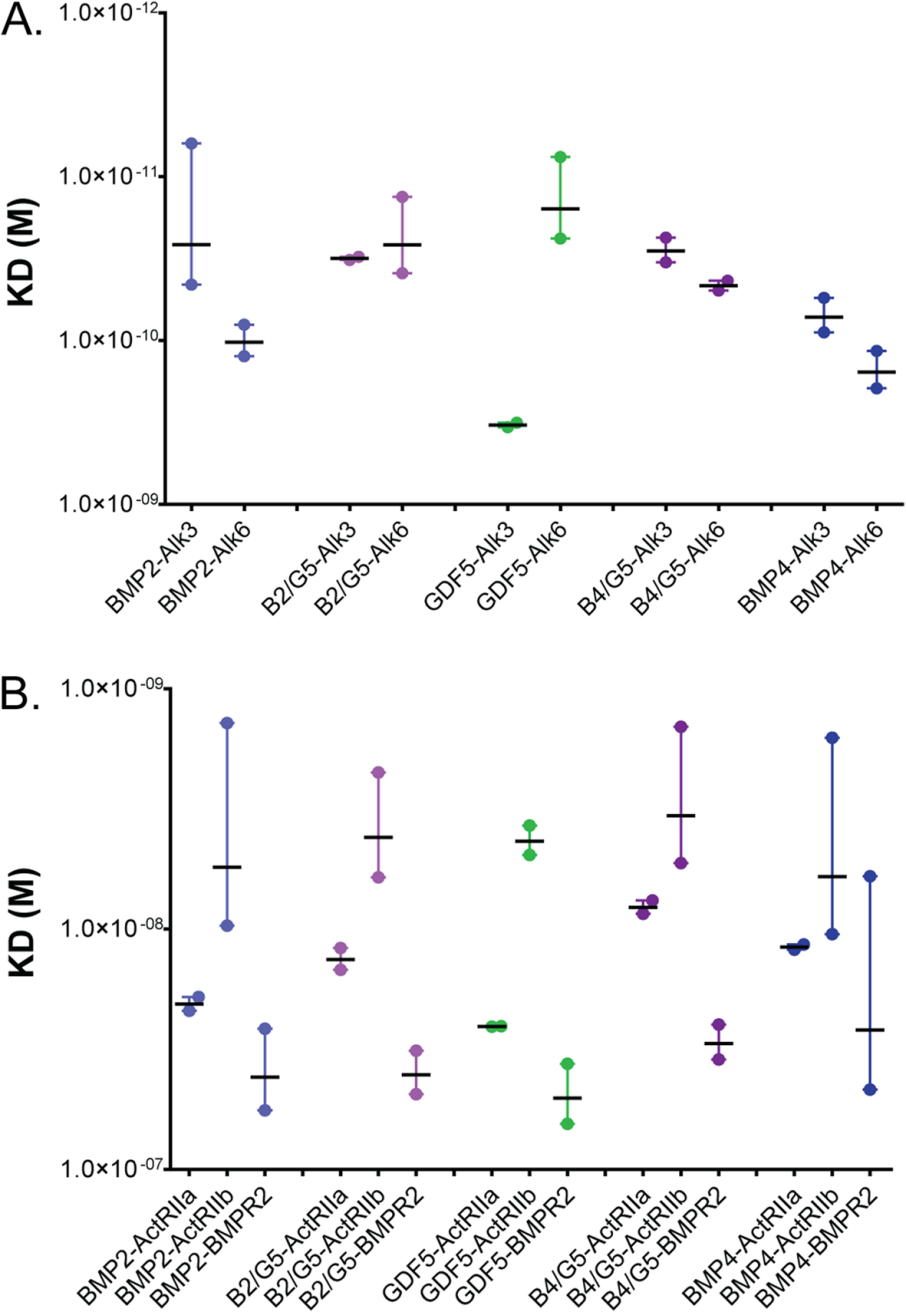
Type 1 and type 2 receptor affinity determined by SPR. **A** Relative binding constants (K_D_) for BMP2 (light blue), BMP4 (dark blue), GDF5 (green), BMP2/GDF5 (light purple), and BMP4/GDF5 (dark purple) to type 1 receptors Fc-(Alk3)_2_ and Fc-(Alk6)_2_, in molarity, as determined by SPR binding curves (Fig. S3) analyzed for kinetic binding using a 1:1 fit to determine association rate (ka) and dissociation rate (kd) for each ligand:receptor pair; K_D_=kd/ka. Reported values are the average of *N*=2 experiments, measured using ligand concentrations between 6.25 nM and 0.045 nM. **B** Binding constant (K_D_) for BMP2 (light blue), BMP4 (dark blue), GDF5 (green), BMP2/GDF5 (light purple), and BMP4/GDF5 (dark purple) to type 2 receptors Fc-(ActRIIa)_2_, Fc-(ActRIIb)_2_, and Fc-(BMPR2)_2_ in molarity, as determined by SPR binding curves (Additional file 4: Fig S4) analyzed for steady state binding. Reported values are the average of *N*=2 experiments, measured using ligand concentrations of 100–0.195 nM (for ActRIIa and BMPR2) or 25–0.195 nM (for ActRIIb)

Differences in receptor binding could be a possible explanation for the observed potency differences between the homo- and heterodimers. In particular, affinity differences might occur given that the composition of the type 1 binding sites is different between the homo- and heterodimers. To test this, we used surface plasmon resonance (SPR) and measured ligand binding to a protein A SPR chip coupled with recombinant chimeric FC-receptors [59]. For the type 1 receptors Alk3 and Alk6, a kinetic analysis was performed to measure binding and fit using a 1:1 binding model. Sensorgrams, along with dissociation and association rate constants, are shown in Additional files 2: Fig. S2 and 3: Fig. S3, and a comparison of the apparent equilibrium dissociation constants (K_D_) is shown in Fig. 5A. As expected, GDF5 displayed a much higher affinity for Alk6 (57 pM) than Alk3 (328pM). BMP4 had a similar affinity for both Alk3 (72 pM) and Alk6 (113 pM), with a mild preference for Alk3. BMP2 preferentially bound to Alk3 (26 pM) but retained the ability to bind to Alk6 (102 pM) as well (Fig. 5A). For all three homodimers, the lower binding affinities were largely driven by an increase in dissociation rates, as the association rates were similar (Figs. S2, S3). Interestingly, both BMP2/GDF5 and BMP4/GDF5 heterodimers displayed high-affinity K_D_ values for both Alk3 and Alk6, with commensurately slow dissociation rates (Figs. 5A, S2, S3). BMP2/GDF5 bound to Alk3 with an apparent affinity of 32 pM and to Alk6 with 26 pM. Similarly, BMP4/GDF5 bound to Alk3 with an apparent affinity of 29 pM and to Alk6 with 46 pM (Fig. 5A). In effect, the heterodimers retained the high affinity of both of their monomeric components, resulting in molecules with a particularly high binding affinity to a larger repertoire of receptors. In addition, we also tested the ability of these proteins to bind to either Alk2 (a preferred target for BMP6 and BMP7) or Alk1 (which binds to BMP9 and BMP10) [24]. As expected, both homodimers and heterodimers displayed similar, almost non-existent affinity to these receptors (Additional file 4: Fig. S4).

Following this, we used SPR to determine the relative binding affinities between BMP ligands and the type 2 receptors. Since the type 2 receptor binding locations are isolated to the individual chains, we hypothesized that the heterodimers would have affinities for the type 2 receptors comparable to the homodimers. For each of the BMP ligands tested, type 2 receptor binding displayed an extremely rapid association followed by an even more rapid dissociation (Additional file 5: Fig. S5A). This rapid dissociation is consistent with the significantly weaker affinity of BMP ligands for type 2 receptors as compared to type 1 receptors, which translates to a more transient interaction. These extremely rapid association and dissociation rates were sufficiently close to asymptotic that the analysis software was unable to fit the data using a kinetic model. Accordingly, these SPR datasets were analyzed for steady-state binding to determine apparent binding affinities. Comparing the different BMP ligands, both homodimeric and heterodimeric proteins displayed a similar affinity for the type 2 receptors, suggesting that the increased potency of BMP2/GDF5 and BMP4/GDF5 as compared to their homodimeric components is not driven by ligand-type 2 receptor interactions (Additional file 5: Fig. S5B).

### Comparison of antagonist inhibition of BMP homodimers and heterodimers

**Fig. 6:**
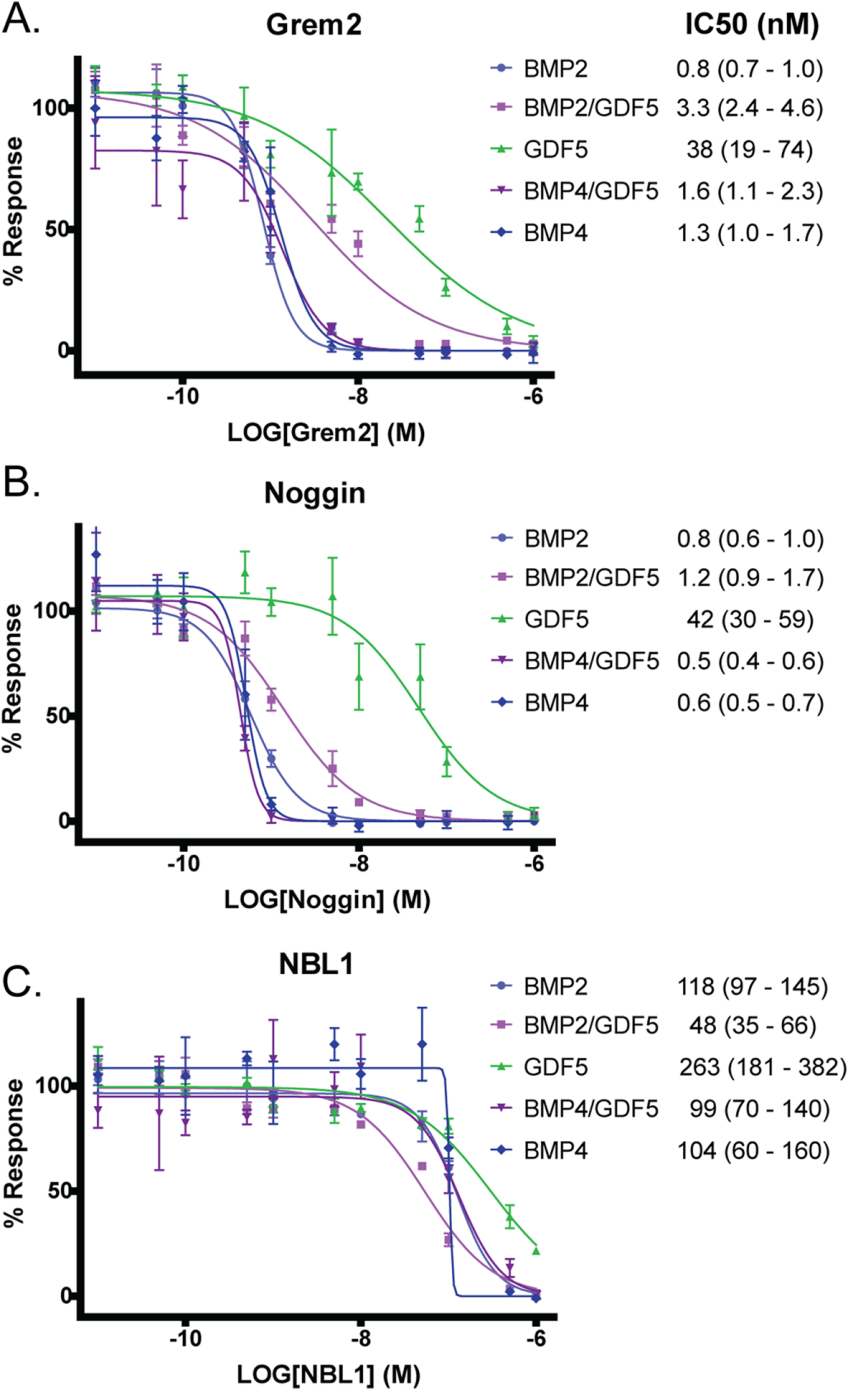
Comparison of inhibitory activity of protein antagonists for heterodimers and homodimers. Inhibition of BMP signaling induced by BMP2 (light blue), BMP4 (dark blue), GDF5 (green), BMP2/GDF5 (light purple), and BMP4/GDF5 (dark purple) by Gremlin 2 (**A**), Noggin (**B**), and NBL1 (**C**), as measured by luciferase reporter assay in BRITER osteoblast cells. Representative experiment shown. Data normalized to untreated control and analyzed using GraphPad Prism using non-linear regression with variable slope and least squares fit. Average IC_50_ of *N*=3 experiments with 95% confidence interval

Since BMP ligands are highly regulated by extracellular protein antagonists, we next wanted to determine if the heterodimers were differentially regulated by BMP antagonists. For instance, it has been shown that the BMP2/BMP7 heterodimer is not neutralized by the extracellular antagonist Noggin, which is known to potently antagonize both BMP2 and BMP7 homodimers [60]. Indeed, a possible explanation for the increased efficacy of the BMP heterodimers, especially in vivo, might revolve around the activity of extracellular antagonists. Accordingly, we tested the effect of several different extracellular protein antagonists, with varying binding modalities and ligand preferences, on BMP signaling using the BRITER luciferase reporter assay. Specifically, we tested Noggin, Grem2, and NBL1. As expected, both Grem2 and Noggin potently antagonized BMP2 and BMP4 at low concentrations, with IC_50_ concentrations of about 1 nM, while requiring much higher concentrations to inhibit GDF5 signaling, with IC_50_s of 38 nM for Grem2 and 42 nM for Noggin (Fig. 6). BMP2/GDF5 and BMP4/GDF5 heterodimers were antagonized by both Noggin and Grem2 at concentrations similar to those needed to inhibit BMP2 and BMP4 (Fig. 6). NBL1 was a relatively poor antagonist for all tested proteins, although it exhibited a slightly higher degree of antagonism towards the heterodimers, particularly BMP2/GDF5 (Fig. 6). These results indicate that both BMP2/GDF5 and BMP4/GDF5 heterodimers are effectively inhibited by extracellular antagonists known to target BMP homodimers, and that differential interactions with these antagonists are not likely contributing to the activity differences observed in cell-culture and in vivo experimental systems. This is important as Noggin in particular is known to be a key regulator of BMP signaling during *Xenopus* development [61, 62].

### Crystal structure of BMP2/GDF5 heterodimer

**Fig. 7:**
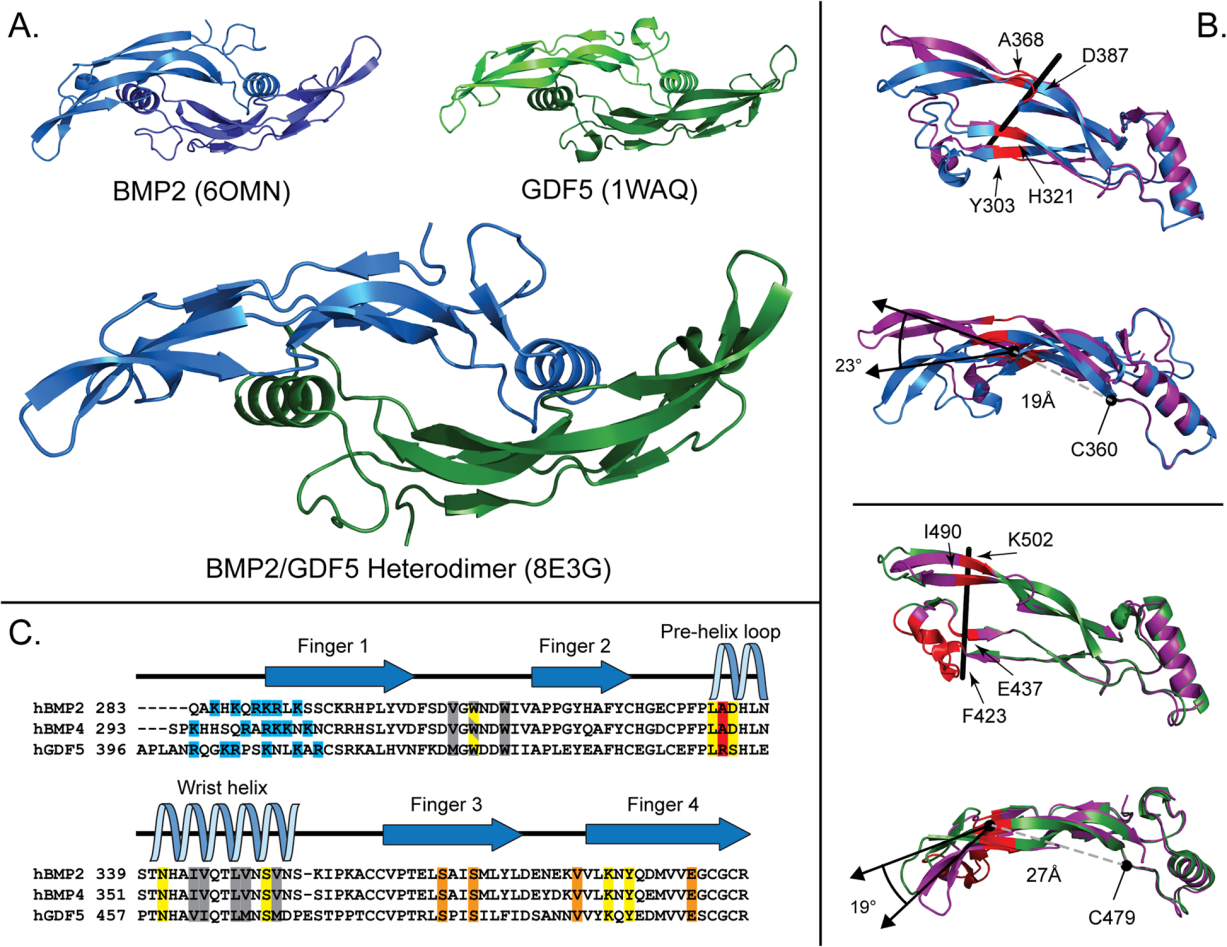
Structure of BMP2/GDF5 heterodimer and comparison of type 1 receptor binding sites to homodimers. **A** Structure of BMP2/GDF5 heterodimer (PDBID: 8E3G) as compared to previously published apo structures of BMP2 (PDBID: 6OMN) and GDF5 (PDBID: 1WAQ) [46, 63]. BMP2 monomer in blue, GDF5 monomer in green. **B** Differences in finger orientation of the BMP2 (Top panel) and GDF5 (Bottom panel) monomers in the heterodimer structure (purple) when compared to their respective homodimeric structures (BMP2 in blue, GDF5 in green) as determined by the DynDom server [64]. Flexible hinge point residues shown in red with the axis of rotation in black. Distance from dimerization cysteine to hinge is annotated in the lower panel of each comparison (gray). **C** Multisequence alignment of the mature domains of human BMP2, BMP4, and GDF5, performed using ClustalOmega [65]. Residues that form hydrogen bonds with type 2 receptors, as determined by PISA analysis of structure of BMP2:Alk3:ActRIIa highlighted in orange (PDBID: 2GOO) [66]. Residues that form hydrogen bonds with type 1 receptors, as determined by PISA analysis of structures of BMP2:Alk3 and GDF5:Alk6 highlighted in yellow (PDBIDs: 1REW, 3EVS) [67, 68]. Conserved hydrophobic residues that form hydrophobic interactions with the type 1 receptor highlighted in gray. Single amino acid difference implicated in Alk3 specificity highlighted in red [26]. Heparin-binding residues highlighted in blue. Secondary structure labeled according to the structure of BMP2 (PDBID: 6OMN) [63]

Our modified oxidative refolding protocol was effective in producing milligram quantities of BMP2/GDF5. Using this protein, we were able to readily solve the X-ray crystal structure of BMP2/GDF5 to a resolution of 2.8 Å (Fig. 7A, Additional file 4: Fig. S4, Additional file 6: Table S1). This afforded us the opportunity to directly cross-compare the structure of the heterodimer to previously solved structures of both the BMP2 and GDF5 homodimers. As expected, the BMP2/GDF5 heterodimer displays the characteristic shape of a BMP growth factor, however, the dimer clearly displays an asymmetrical appearance (Fig. 7A). This is in stark contrast with all published structures of BMP ligands, both apo structures and complex structures containing other protein binding partners, which retain the same basic symmetrical shape. As such, in a number of previous structures the asymmetric unit only contains one half of the ligand. Here, due to the asymmetry of the ligand, the asymmetric unit consists of two full dimers. The RMSD for the two dimers is 0.28 for 171 Cα indicating that crystal packing, which is different for each dimer, has little impact on the overall shape of each ligand.

A significant difference between the heterodimer and homodimer structures occurs in the curvature of the fingers. Compared to the apo-BMP structure, the BMP2 half of the heterodimer displays much less curvature in the β-strands that form the “fingers” of the BMP fold. When analyzed using DynDom, which can be used to measure differences in domain orientation between structures, the heterodimer structure shows a 23° decrease in curvature with a hinge point located in the “finger” region, measured at 19 Å from the dimerization disulfide cysteine (Fig. 6B) [64]. In contrast, the GDF5 half exhibits slightly increased curvature of the β-strands compared to apo-GDF5 (Fig. 6B). Here, the point of divergence (the “hinge”) occurs very close to the tips of the “fingertips,” 27 Å from the central disulfide bond, minimizing the number of residues that differ from the apo-GDF5 structure. Thus, the fingers of the heterodimer can be described as opening on the BMP2 side and closing on the GDF5 side. This overall difference in curvature in the fingers across the dimer is responsible for the asymmetrical shape of the growth factor. To aid in the discussion of these different sites we will name the type 1 site with the BMP2 fingers and the GDF5 wrist as B_F_G_W_ and vice versa, G_F_B_W_, for the other side (Fig. S6).

### Analysis of the surface electrostatics

**Fig. 8:**
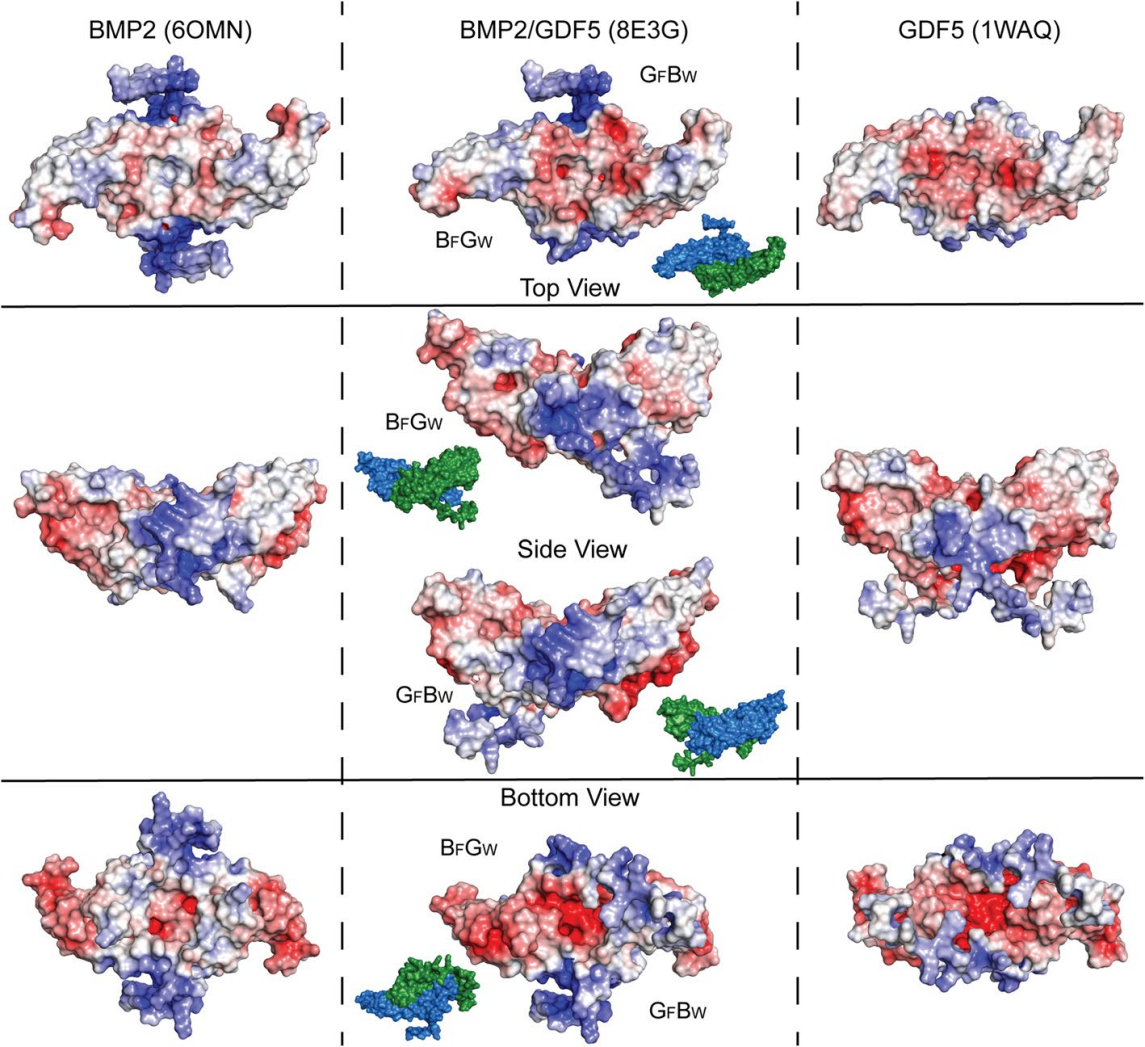
Electrostatic comparison of BMP2/GDF5 to respective homodimers. Surface representation of homodimeric BMP2 (6OMN), GDF5 (1WAQ), and BMP2/GDF5 heterodimer (8E3G) colored by solvent electrostatic potential as determined by Adaptive Poisson-Boltzmann Solver (APBS) plugin in PyMol [46, 63, 69]. Residues not represented in the PDB deposition were modeled based on AlphaFold models of BMP2 and GDF5 [70]. Electrostatic scale ranges from -5 (red) to 5 (blue) k_B_Te_c_^−1^. Top, bottom and side views are displayed, including both asymmetrical sided of the BMP2/GDF5 heterodimer

The disparity in heparin/HS binding affinity between the BMP2/GDF5 heterodimer and the BMP2 and GDF5 homodimers was initially observed in our protein production protocol, where heparin affinity chromatography is used to separate the heterodimer from the homodimers. This difference can be visualized when comparing the solvent electrostatic potential of the homodimer and heterodimer structures. Here, the positively charged surfaces (colored blue) correspond to clusters of positively charged residues, including arginine and lysine, and are closely associated with heparin/HS binding regions (Fig. 8). The BMP2 HS binding site has been definitively mapped to a patch of lysine and arginine residues (QAKHKQRKRLK-) located the N-terminus of the mature polypeptide, at the base of the protein facing the cell (Figs. 7 C and 8, bottom view) [31]. Both BMP4 and BMP2 have a continuous length of three basic amino acids, which in the case of BMP4 was demonstrated to be the key driver of heparin binding (Fig. 7C) [71]. GDF5 possesses a similar number of lysine and arginine residues to BMP2 and BMP4, but they are spread out over a longer N-terminal extension, leading to a lower local concentration of positive charge (Fig. 7C). Additionally, GDF5 is extremely negatively charged at the base of the dimer, which likely offsets the effect of the smaller positive patches that can be observed in the front view of the surface (Fig. 8). GDF5’s lower heparin binding affinity results in reported mammalian serum levels 10-20 fold higher than those of other BMPs [72–74]. Using the crystal structure, we analyzed the electrostatic surface of the BMP2/GDF5 heterodimer. This analysis revealed that the BMP2/GDF5 heterodimer displays a more neutrally charged surface at that site and retains enough positively charged residues from its BMP2 half to form a much more limited heparin/HS binding motif, consistent with its observed intermediate heparin binding affinity (Figs. 1 and 8C).

### Analysis of type 1 binding sites in the BMP2/GDF5 structure

Previous structures have illuminated how both Alk3 and Alk6 bind to BMP2 and GDF5, respectively [52, 68]. There are many similarities between these two ligand:receptor complex structures, with conserved binding locations and hydrophobic interactions dominating the interfaces, including the hydrophobic triad located at the concave finger regions of one monomer and the face of the wrist helix of the second (Fig, 7C). In addition, similar hydrogen bonds also promote receptor binding (Fig. 7C). One key hydrogen bond is formed between the backbone of L333 of BMP2 and Q109 of Alk3, which when perturbed by mutation to proline, results in ablated receptor binding and signaling [75]. This specific hydrogen bond is also apparent in the structure of GDF5 bound to Alk6, although its necessity has not been experimentally validated [68]. These similarities in receptor-binding interfaces are expected as BMP2 signals through both Alk3 and Alk6, largely interchangeably. However, one key difference, and the reason that GDF5 has a noticeably lower affinity for Alk3 than Alk6, is a difference between the residue immediately following this key hydrogen bond at L333 (L451 in GDF5), which is Ala in BMP2 and Arg in GDF5. Mueller and colleagues showed that R452, which is located in the wrist of GDF5 (A334 in BMP2), is the major factor that allows GDF5 to discriminate Alk6 over Alk3, likely due to steric clashes with Alk3 [26]. The BMP2/GDF5 heterodimer possesses two unique type 1 receptor binding sites, B_F_G_W_ with this inhibitory Arg located in the wrist helix and G_F_B_W_ with the more permissive Ala (Additional file 7: Fig. S6). Thus, the B_F_G_W_ binding site will likely preferentially bind to Alk6 over Alk3. Indeed, superposition of both Alk3 and Alk6 into this site shows that R438 would sterically clash with Alk3, much like in homodimeric GDF5. However, due to the differences in curvature of the fingers, the type 1 site is more open which shifts several hydrophobic residues away from the superimposed receptor. Thus, while the B_F_G_W_ site maintains the discriminatory R438 residue of GDF5, the overall shape of the pocket is more open, which might allow the receptors to position themselves slightly differently than that observed for the homodimers. The G_F_B_W_ binding site is likely to function similarly to homodimeric BMP2, with a preference for Alk3 over Alk6 and the ability to signal strongly through both receptors.

We next attempted to gain experimental information about the receptor specificity of each unique site in the heterodimer and to investigate the possibility of increased Alk3 affinity in the B_F_G_W_ binding pocket. To do this, we took advantage of the BMP2 mutation, L333P, that disrupts type 1 receptor binding in order to disrupt receptor binding on one half of the heterodimer. First, the BMP2 homodimer with the L333P mutation was produced as a control and shown to not signal in a luciferase reporter assay or bind Alk3 or Alk6 by SPR (Additional file 8: Fig. S7 and Additional file 9: Fig. S8). Using the L333P BMP2 we formed and isolated a heterodimer with WT GDF5, which would be predicted to have a disrupted type 1 receptor binding on one half (G_F_B_W_), leaving a single functional site on the other side (B_F_G_W_). The mutant heterodimer displayed a 10-fold reduction in signaling activity when compared to the WT heterodimer, but only a 2-fold reduction when compared to GDF5 using the BRITER cell reporter assay (Additional file 8: Fig. S7). Analyzing the mutant heterodimer by SPR also showed a reduction in receptor affinity (Additional file 9: Fig. S8). Using a 1:1 binding model, the mutant heterodimer, BMP2_L333P/GDF5, binds to both Alk3 and Alk6 with much lower affinity than homodimeric GDF5, consistent with the mutant only possessing a single type 1 receptor binding site. While it is difficult to differentiate the effects of avidity vs affinity in this binding system, it should be noted that the relative decrease in binding is similar for both Alk3 vs Alk6 binding (approximately a 5-fold decrease in K_D_ when comparing BMP2_L333P/GDF5 to WT GDF5 homodimer). Additionally, using a bivalent binding model reveals the initial binding events are almost identical between homodimeric GDF5 and heterodimeric BMP2_L333P/GDF5 for both Alk3 and Alk6 (Additional file 9: Fig. S8). This is consistent with functionally identical type 1 binding sites. This implies that at least in this experimental system, the decreased curvature of the BMP2 fingers does not have a measurable impact on binding, lending support to the binary complex modeling we performed. Accordingly, the increased overall affinity for the type 1 receptors by the heterodimers is likely due to having two different high-affinity sites packaged together in one signaling ligand, with one side behaving like a GDF5 ligand and the other behaving like a BMP, with specificity dictated by the wrist region. Unfortunately, we were unable to replicate these findings by disrupting the B_F_G_W_ site, as GDF5 mutations did not properly refold.

## Discussion

In recent years, there has been an increased interest in the study of heterodimeric growth factors in the TGFβ superfamily. These proteins, unlike the better-studied homodimers, could serve as asymmetric signaling platforms that target a wider array of receptors to induce signaling on a broader level by combining features of both “parent” monomers as well as novel features [35–37]. Unfortunately, only a relatively few papers have been published on a handful of BMP heterodimers. This is in part due to the difficulty in producing and purifying recombinant heterodimeric proteins, and in the difficulty of isolating heterodimer-specific activity from that of homodimer in model systems. Accordingly, at the molecular level, relatively little is known about the general mechanism of BMP heterodimer function, which structural elements are key to their unique activities, and even which of the numerous “potential” heterodimers might exist naturally or can be artificially synthesized.

In order to extend the study of BMP heterodimers into novel proteins, specifically ones containing members of the GDF5/6/7 subclade which have not been previously studied, we synthesized recombinant BMP2/GDF5 and BMP4/GDF5 through the oxidative refolding of bacterially produced mature proteins (Fig. 1). We have demonstrated that these heterodimers possess unique biochemical and biological properties. Indeed, we have established that both heterodimers are more potent signaling molecules than their corresponding homodimeric proteins, in both in vitro and in vivo experimental systems. It should be noted that all these experiments were performed using recombinant protein produced in *E. coli*. There is evidence that some bacterially produced BMPs are less potent than when produced in mammalian cells, including commercially available BMP2 and BMP4 [75–77]. However, as all these proteins are bacterially produced, the direct comparison between homodimer and heterodimer activity is valid.

This study represents the first structure of a TGFβ heterodimer, which highlights the asymmetrical receptor binding pockets and curvature of the BMP2/GDF5 heterodimer. Interestingly, these differences have not been observed across the current apo structures of any BMP ligands, not simply those of BMP2 and GDF5. This asymmetric structure allows the heterodimers to bind with a combined higher affinity to both Alk3 and Alk6 than either of the homodimers, which are more optimized for a single type 1 receptor. This is consistent with an increase in heterodimer signaling potency when compared to their component homodimers, driven by a high affinity for a wider array of type 1 receptors. Since the affinities for type 2 receptors and susceptibility to antagonists is similar between the homo- and heterodimers, the observed potency differences are not derived from these interactions.

The importance of studying protein activity across a range of model types is highlighted by comparing the differences seen here between our in vitro and in vivo systems. In the BRITER luciferase reporter assay system, all homodimeric BMPs induced signaling at similar rates, with EC_50_ around 4–5 nM. However, BMP2 had a much higher maximum signal than either GDF5 or BMP4. The heterodimers induced signaling at lower concentrations than the homodimers, with EC_50_s ~1.5 nM, and also demonstrated higher maximum signaling (Fig. 2). This could be explained by the relatively higher average affinity to both Alk3 and Alk6 of the heterodimers and, to a lesser extent, BMP2 (Fig. 5).

In more complex in vivo systems, which offer a more complete model of protein biology, different homodimeric BMPs had differential potencies. In a *Xenopus* development assay, GDF5 signaled much more potently at lower concentrations than did BMP2 or BMP4 (Fig. 3). While this could potentially be explained by differences in receptor expression between assay systems, previous research has shown that *Xenopus* blastula express roughly equivalent levels of Alk3 and Alk6 between stages NF7-NF37 [78]. Other possible explanations are the presence of extracellular antagonists such as Noggin in the system, and the presence of a complex extracellular matrix comprised partially of heparin sulfate proteoglycans [61, 62, 79]. GDF5 is partially resistant to Noggin antagonism (Fig. 6) and possesses a lower affinity to heparan than BMP2 or BMP4 (Fig. 1), which could mean that it diffuses more widely and is more available for longer-term signaling [72–74]. In effect, GDF5 may have the ability to serve as more of an endocrine signaling molecule, as opposed to BMP2 and BMP4. This is supported by the in situ hybridization data, performed on samples collected only a few hours post-injection showing a much broader range of signaling for GDF5, which agrees with the results of the DAI scoring (Fig. 3). Interestingly, in this system, the heterodimers induce signaling at levels similar to GDF5, in spite of a lack of protection from Noggin antagonism and an intermediate heparan affinity. Thus, the presence of the GDF5 chain both decreases heparin binding and increases total receptor affinity. In contrast, in an in vivo assay system using zebrafish, the BMP2/GDF5 heterodimer signals more potently than either homodimeric BMP2 or GDF5 while BMP2 induces signaling much more robustly than GDF5. In this system, the proteins are tested at a much more biologically relevant concentration, and over a much shorter time scale, likely mitigating any diffusion-induced effects.

## Conclusions

In summary, we have developed novel BMP2/GDF5 and BMP4/GDF5 heterodimers which signal more potently than homodimeric BMP2, BMP4. or GDF5 across an array of in vitro and more complex in vivo assays. Interestingly, in each of these experimental systems, the tested heterodimers functioned similarly to the most potent homodimer, BMP2 in zebrafish signaling assay and GDF5 in *Xenopus* development. In each case, the heterodimer retains the function of its monomeric component most suited to high activity. The evidence presented here indicates that this is likely due, at least in part, to a high affinity of the heterodimers to multiple different type 1 receptors. While this is likely the main driver of potency, the heterodimers also display differences in heparin affinity and may retain a higher diffusion potential similar to GDF5.

One advantage of developing these techniques for recombinant heterodimer formation is that it allows one to modify each individual chain to generate a spectrum of ligands. Initially proposed in the laboratory of Senyon Choe, these bacterial heterodimers can be used to generate asymmetrical signaling molecules with specifically tailored properties, say a single chain with specific receptor affinity or resistance to a specific antagonist. These specifically tailored heterodimers could then be used as a biochemical tool to interrogate the importance of bivalent interactions in TGFβ biology: whether a hetero-receptor complex displays alternative biology or determining the importance of avidity interactions for different protein antagonists. In addition, as recombinant BMPs are utilized more and more frequently as therapeutics, a more precisely tunable signaling platform could be important for targeting specific biological functions.

In addition to serving as biochemical tools and as potential new therapeutics, further work is needed to determine whether these BMP/GDF heterodimeric proteins are biologically relevant. Given the TGFβ family consists of approximately 30 genes, a combinatorial analysis proposes the possibility of over 500 potential heterodimeric proteins. The generation of artificial recombinant ligands should help determine whether these specific BMP/GDF heterodimers, and which of the other 500+ possible heterodimers, naturally occur, and what their biological roles may be. The platform described in this paper can aid in the very challenging aspects of heterodimer identification and functional characterization.

## Methods

### Protein Production

The cDNA constructs for mature BMP2 (received from Hongwen Ma), BMP4, and GDF5 were cloned into the pET21a bacterial expression vectors and transformed into Rosetta (DE3) *E. coli*. The BMP2 L333P mutant was generated by performing overlapping primer PCR on the BMP2 construct. Transformed bacteria were grown to log phase and induced with 1 mM IPTG. After 18h, the bacterial cultures were pelleted and sonicated to isolate the inclusion bodies, which were then washed with PBS with 0.1 % Triton X and solubilized in 8 M urea, 100 mM DTT, 100 mM TRIS and 1 mM EDTA (pH 8.5). Solubilized inclusion bodies were dialyzed into 4 M urea, 100 mM TRIS, and 1 mM EDTA acidified to pH ~3.0 with glacial acetic acid. Dialyzed samples were then clarified by centrifugation to isolate soluble BMP from insoluble contaminating proteins and visualized by SDS-PAGE to ensure a minimum of 90 % purity. BMP2 or BMP4 was then mixed with a molar equivalent of GDF5 and diluted dropwise to a final concentration of 0.1 mg/mL in an oxidative refolding buffer containing 0.1 M Tris pH 8.5, 0.5 M L-Arginine, 0.3 % CHAPS, 150 mM NaCl, 1 mM reduced glutathione, 1 mM oxidized glutathione, 1 mM EDTA [46, 47]. After 5 days, the refolded protein was diluted 1:2 in urea to a final concentration of 4 M, HCl added to adjust the pH to ~5, and loaded onto a heparin affinity column. Protein was then eluted with a NaCl gradient from 150 to 650 mM over 30 column volumes to separate out homodimers from heterodimer by variable heparin affinity. Purity was confirmed with SDS-PAGE, western blot, and MALDI-TOF mass spectroscopy (UC-COM Proteomics Core). This protocol was modified from those developed for the production of BMP2 and GDF5 by oxidative refolding of bacterially produced protein in inclusion bodies [46, 47]. Gremlin-2 and NBL1 were produced as previously described [80, 81]. Recombinant Noggin was purchased from Peprotech.

All recombinant proteins produced for this manuscript were quantitated by measuring the A280 on a spectrophotometer, corrected using the theoretical extinction coefficient (as calculated by Expasy ProtParam). These values were then confirmed by SDS-PAGE stained with colloidal Coomassie and BCA assay. Upon quantitation, all proteins were flash-frozen in small aliquots and thawed as needed for experiments.

### Western blots

Protein samples were subjected to SDS-PAGE under both reducing and non-reducing conditions, to detect both the dimer and its component monomers. Gels were then incubated in a buffer containing the reducing agents BME and DTT to reduce the proteins in gel, which increases consistency in antibody binding, before being transferred to nitrocellulose membranes. Membranes were then probed with anti-BMP2 (rabbit IgG, Fitzgerald 70R-BR001), anti-BMP4 (rabbit IgG, Invitrogen PA5-78875), or anti-GDF5 (goat IgG, R&D AP-853). Secondary antibodies used were mouse anti-rabbit IgG-HRP (Santa Cruz sc-2357) and horse anti-goat IgG-HRP (Vector PI-9506).

### Cell culture

BRITER cells, a BMP2 and BMP4 deficient murine osteoblast cell line stably transfected to produce luciferase dependent on a SMAD1/5/9 activated BRE promoter (a gift from Amitabha Bandyopadhyay), were grown in MEM α supplemented with 10% FBS, 100 μg/mL Hygromycin B and 1X Penicillin/Streptavidin [48]. Cells were passaged every 3-4 days, split 1:10, and grown under culture conditions at 37 °C with 5% CO_2_.

### Luciferase reporter assays

BRITER cells were plated in a 96-well microplate at a concentration of 20,000 cells/well and allowed to attach for 18 h. Growth media was then replaced with serum-free Dulbecco’s modified essential media (Corning, 10-017-CV) and cells were starved for 5 h. For excitation assays, cells were then treated with exogenous BMP protein serially diluted (from 500 nM to 0.049 nM) in DMEM. For inhibition assays, inhibitory proteins were diluted (from 1 μM to 0.1 nM) in DMEM with a constant amount of ligand (1 nM BMP2, 1 nM BMP2/GDF5, 1 nM BMP4/GDF5, 5 nM BMP4, 5 nM GDF5). After 3 h, cells were lysed, added to luciferin reagent (Promega, E1501), and luciferase activity was determined by measuring luminescence over 10 s with a Biotek Synergy H1 plate reader using Gen5 software. Data were normalized to untreated controls, plotted using GraphPad Prism, and analyzed by non-linear regression. All experiments were performed in triplicate. Reported values are an average of *N*=3 separate experiments.

### Surface plasmon resonance

Binding kinetics of BMP growth factors to their high-affinity type 1 receptors were determined by surface plasmon resonance using a BIAcore T-200 optical sensor system (GE Healthcare) and analyzed using BIAevaluation 4.1 software as previously reported [44]. In brief, chimeric FC-(Alk6)_2_ (R&D, Cat#: 505-PR-100), FC-(Alk3)_2_ (R&D, Cat#: 315-BR-100/CF), FC-(Alk1)_2_ (R&D, Cat#: 370-AL-100), or FC-(Alk2)_2_ (R&D, Cat#: 637-AR-100) were coupled to a Series S Protein A chip (Cytiva, 29127556). Purified homodimeric and heterodimeric BMP proteins, serially diluted in SPR buffer (20 mM HEPES pH 7.4, 350 mM NaCl, 0.005 % P-20, 0.5 mg/mL BSA, 3.4 mM EDTA) from a concentration of 6.25–0.049 nM, were flowed over the chip at 50 μL/min for 300 s to determine association, then washed off for 1000 s to determine dissociation. Type 1 receptor binding data was analyzed for kinetic binding using a 1:1 fit, as an average measure for receptor affinity for a single ligand, or using a bivalent fit, to distinguish between the initial binding event and the 2^nd^ binding event to the 2^nd^ binding site on the dimer.

Binding of the BMP growth factors to their lower affinity type 2 receptors is too transient to be successfully analyzed using a kinetic fit, thus affinity was measured by steady-state interactions. Chimeric FC-(ActRIIa)_2_ (R&D, Cat#: 340-R2-100/CF), FC-(ActRIIb)_2_ (R&D, Cat#: 339-RB-100/CF), or FC-(BMPR2)_2_ (R&D, Cat#: 811-BR-100) were coupled to a Series S Protein A chip (Cytiva, 29127555). Purified homodimeric and heterodimeric BMP proteins, serially diluted in SPR buffer from a concentration of 100–0.198 nM, were flowed over the chip at 50 mL/min for 300 s to determine association, then washed off for 1000 s to determine dissociation.

### *Xenopus* development assays

Stage 9 *Xenopus laevis* blastula, >55 per condition, were microinjected into the blastocoel with different concentrations of purified BMP ligand homodimers or heterodimer: 0.05 pmol in 4.3 nL, 0.15 pmol in 13nL, 0.5 pmol in 40nL, 1.5 pmol in 120nL or with 10mM HCl vehicle control at the same volumes. Dose-response experiments measuring embryo survival were performed to determine the minimal effective dose of BMP ligand homodimers or heterodimers for subsequent biological assays (Fig. 3A). Embryos were permitted to develop until stage NF37, and then blindly scored using the dorso-anterior index (DAI) to determine the extent of ventralization caused by excessive BMP signaling [49, 82]. In the DAI scoring system, 5 is a normally developed tadpole and increasing ventralization corresponds with a progressively lower score down to 0 for a fully ventralized blastula (Fig. 3) [49]. Injected *Xenopus* blastula were compared to uninjected controls. DAI scoring is reported for the 0.15 pmol and 0.5 pmol doses.

An additional 20 embryos per condition were injected and fixed at gastrula stage NF10, bisected, and assayed by in situ hybridization to determine the expansion of *ventx1/2* expression, which are well-known direct BMP/SMAD1 target genes as described previously [82, 83]. *In situ* hybridization results are reported for embryos injected with 0.15 pmol and 0.05 pmol doses. All *Xenopus* experiments were performed in compliance with ethical regulations outlined by the NIH and institutional guidelines under Cincinnati Children's Hospital Medical Center Institutional Animal Care and Use Committee (IACUC) approved protocol IACUC2022-0026, approved 7/22/2022.

Experiments with <50% survival of control embryos were excluded. DAI scoring experiments were performed a minimum of 3 independent times while in situ hybridization staining was performed once. Data were analyzed by 1W-ANOVA or Chi-square test in Prism 9 (GraphPad). Graphs were created in Prism 9.

### Zebrafish signaling assay

*Bmp7a* ^*sb1aub*^ mutant Zebrafish embryos at 3 hpf were injected with 0.08 fmol of recombinant BMP2 homodimer, GDF5 homodimer, or BMP2/GDF5 heterodimer protein. After 30 min, embryos were fixed in 4% PFA PBS, and subjected to immunohistochemistry to probe for pSMAD5 to quantify nuclear pSMAD5 as previously described [57]. The pSMAD5 antibody was obtained from Cell Signaling Technology (#13820). Imaging was performed using a Zeiss LSM 880 confocal microscope with a LD LCI Plan-Apochromat 25×/0.8 Immersion Corr DIC M27 multi-immersion lens. Quantification of the nuclear pSMAD5 fluorescence in embryos was done by calculating the average intensity of the brightest 75% of nuclei minus the average intensity of the dimmest 25% (assumed background). Signaling was compared to uninjected WT and uninjected mutant controls and also analyzed for nuclear pSMAD5 by immunohistochemistry.

### Crystallography

Crystals were produced using the hanging drop method. Purified BMP2/GDF5 heterodimer, whose identity had been confirmed by mass spectrometry, was concentrated to 12.4 mg/mL in 10 mM HCl, and then screened for crystallization conditions. Optimized crystals were grown in 100 mM Tris (pH 8.0) with 0.5 M Mg formate, harvested, cryo-protected in 200 mM HEPES (pH 7.5) with 4 M Mg formate, and frozen in liquid nitrogen. X-ray diffraction data were collected with an Eiger 16M detector at the GM-CA beamline 23-ID-B of the Advanced Photon Source at Argonne National Laboratories. Diffraction data were indexed using iMosfilm, scaled with Aimless, and phases were generated using molecular replacement searching with the previously solved structures of BMP2 (1REU) and GDF5 (1WAQ), using PhaserMR [26, 63, 67, 84–86]. Final refinement was performed with Phenix Refine and REFMAC5, and modelbuilding was done in Coot and validated using Molprobity [87–90].

The structure was submitted to PDB-REDO for validation [91]. As an additional form of internal validation, models of homodimeric BMP2, homodimeric GDF5, heterodimeric BMP2/GDF5 with the chain positions swapped, and heterodimeric BMP2/GDF5 with the chains in the final solved positions were generated using the final structure as a location framework and the BMP2 and GDF5 monomeric structures from 6OMN and 1WAQ, respectively [26, 63]. These models were used to phase the initial mtz file and then subjected to a single, identical refinement step. While all models were successfully phased, upon refinement there was a sharp divergence whereby the heterodimeric structure with the BMP2 and GDF5 chains placed in their final solved position (as opposed to their positions inverted), resulted in R_work_ and R_free_ scores 3% better than any of the other tested models. These validation steps gave us confidence in our final model. The Ramachandran outliers observed are consistent with previously solved crystal structures of BMP2 and GDF5 [26, 67].

### Structural analysis and modeling

Dynamic regions of the BMP2/GDF5 heterodimer structure (8E3G) were compared to homodimeric BMP2 (6OMN) and homodimeric GDF5 (1WAQ) using the DynDom server [26, 63, 64]. Type 1 receptor binding was modeled by aligning the heterodimer structure to specific monomers of published binary structures of BMP2:Alk3 (1REW) and GDF5:Alk6 (3EVS) in PyMol [67, 68, 92]. Models were produced of Alk3 and Alk6 interacting with each unique type 1 binding site at the heterodimer, with the receptor placement determined by aligning to the wrist helix interface of each binary structure. Additional models of BMP2:Alk6 and GDF5:Alk3 interactions were produced in the same way. All models were analyzed using the PISA webserver for predicted interface stability and binding energy [93, 94]. For appropriate electrostatic surface mapping, the additional N-terminal residues that comprise the heparin-binding domains of BMP2 and GDF5, which do not appear in density for any solved structure, were modeled in as unstructured loops using the AlphaFold models of BMP2 and GDF5 [70]. Electrostatic surface representation was then generated by APBS [69].

## Supplementary Information

**Additional file 1: Figure S1.** Validation of heterodimer identity and purity by mass spectrometry. Mass of purified protein peaks separated by heparin affinity chromatography validated by MALDI-TOF mass spectrometry. Measured molecular weight (MW) within permitted error of theoretical MW for pure homodimeric or heterodimeric proteins. Note: our construct of BMP4 contains an additional Met residue on the N-terminus of the protein, which impacts the predicted molecular weight of BMP4 homodimer, BMP4/GDF5 and monomeric BMP4 accordingly.

**Additional file 2: Figure S2.** Representative SPR kinetic binding curves for ligand interactions with type 1 receptors. Representative plots of SPR binding curves of homodimeric and heterodimeric ligands to type 1 receptor Fc chimeras. Experimental traces (black) were fit using a 1:1 binding model and the fits are represented as a red line. All experiments were performed with variable ligand concentrations between 6.25 nM – 0.045 nM.

**Additional file 3: Figure S3.** Calculated association and dissociation binding constants for ligand interactions with type 1 receptors, determined by kinetic SPR. Average of *N*=2 experiments.

**Additional file 4: Figure S4.** Representative SPR kinetic binding curves for ligand interactions with more type 1 receptors. Representative binding curves of BMP2 binding to Alk2 and Alk1, measured by SPR. All tested growth factors bound similarly to these receptors. All experiments were performed with variable ligand concentrations between 6.25 nM – 0.045 nM.

**Additional file 5: Figure S5.** Steady-state analysis for ligand interactions with type 2 receptors. A) Representative T2 binding curve, GDF5 and (ActRIIB)_2_-Fc, and Steady-State binding fit. B) Table of binding constants (K_D_, in nM) of homodimeric and heterodimeric growth factors to type 2 receptors, determined by steady state analysis. Average of N=2 experiments. All experiments were performed with variable ligand concentrations between 100 nM – 0.195 nM (for ActRIIA and BMPR2) or 25 nM – 0.195 nM (ActRIIB).

**Additional file 6: Table S1.** X-ray diffraction data and refinement statistics.

**Additional file 7: Figure S6.** Comparison of type 1 receptor binding pockets of BMP2, GDF5, and BMP2/GDF5 heterodimer. The Alk3 and Alk6 extracellular domains from binary complex structures 1REW and 3EVS, respectively, were aligned (using PyMol) to homodomeric BMP2 (6OMN), GDF5 (1WAQ) or BMP2/GDF5 heterodimer (8E3G), based on the alignment of the ligands targeting the wrist helix of binding pocket [46, 63, 92]. Residues within 5Å of receptors colored yellow. Leucine required for type 1 binding (L333 in BMP2, L451 in GDF5) in cyan [67]. Residue implicated in Alk3 vs Alk6 binding preference in GDF5 in red (A334 in BMP2, R452 in GDF5) [26].

**Additional file 8: Figure S7.** Luciferase reporter assay for mutant heterodimer. Luciferase Reporter Assay used to compare function of BMP2/GDF5 heterodimer (purple) to BMP2 homodimer (blue), GDF5 homodimer (green), or a combination of BMP2 and GDF5 homodimers (gray). Representative curves shown. Data normalized to untreated control and analyzed using GraphPad Prism using non-linear regression to determine EC_50_. Data tables display an average of *N*=3 experiments, with 95 % confidence range reported.

**Additional file 9: Figure S8.** SPR kinetic binding curves for mutant heterodimer. SPR binding results for GDF5, BMP2_L333P, and BMP2_L333P/GDF5 heterodimer, analyzed for kinetic binding using both a 1:1 binding model and a Bivalent binding model. All experiments were performed with variable ligand concentrations between 6.25 nM – 0.045 nM.

## Data Availability

The atomic coordinates for the crystal structure presented in this work are available in the RCSB Protein Data Bank, under the code 8E3G All other primary data and materials produced for this project will be made available upon request by the corresponding author.

## Abbreviations

TGFβ: Transforming growth factor β
BMP: Bone morphogenetic protein
DAI: Dorso-anterior index
hpf: Hours post fertilization
SPR: Surface plasmon resonance
APBS: Adaptive Poisson-Boltzmann Solver
HS: Heparan sulfate
