# Supplementary figures and images for: Formation and characterization of BMP2/GDF5 and BMP4/GDF5 heterodimers
Source: BMC Biol. 2023 Feb 1;21:16. doi: 10.1186/s12915-023-01522-4 (PMC9893541; doi:10.1186/s12915-023-01522-4)

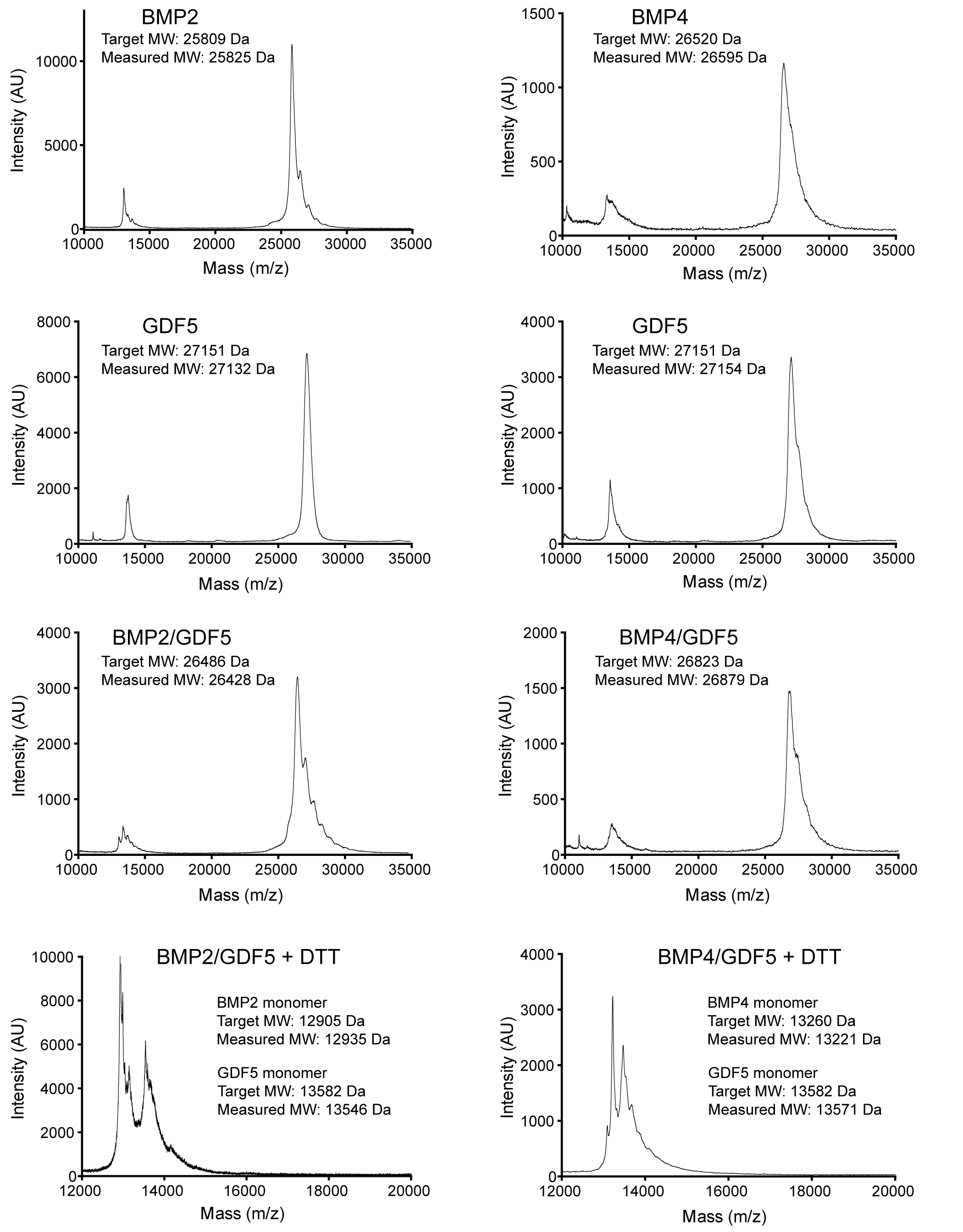

Supplement: Supplementary file 1 — Additional file 1: Figure S1. Validation of heterodimer identity and purity by mass spectrometry. Mass of purified protein peaks separated by heparin affinity chromatography validated by MALDI-TOF mass spectrometry. Measured molecular weight (MW) within permitted error of theoretical MW for pure homodimeric or heterodimeric proteins. Note: our construct of BMP4 contains an additional Met residue on the N-terminus of the protein, which impacts the predicted molecular weight of BMP4 homodimer, BMP4/GDF5 and monomeric BMP4 accordingly. [file 12915_2023_1522_MOESM1_ESM.tif]

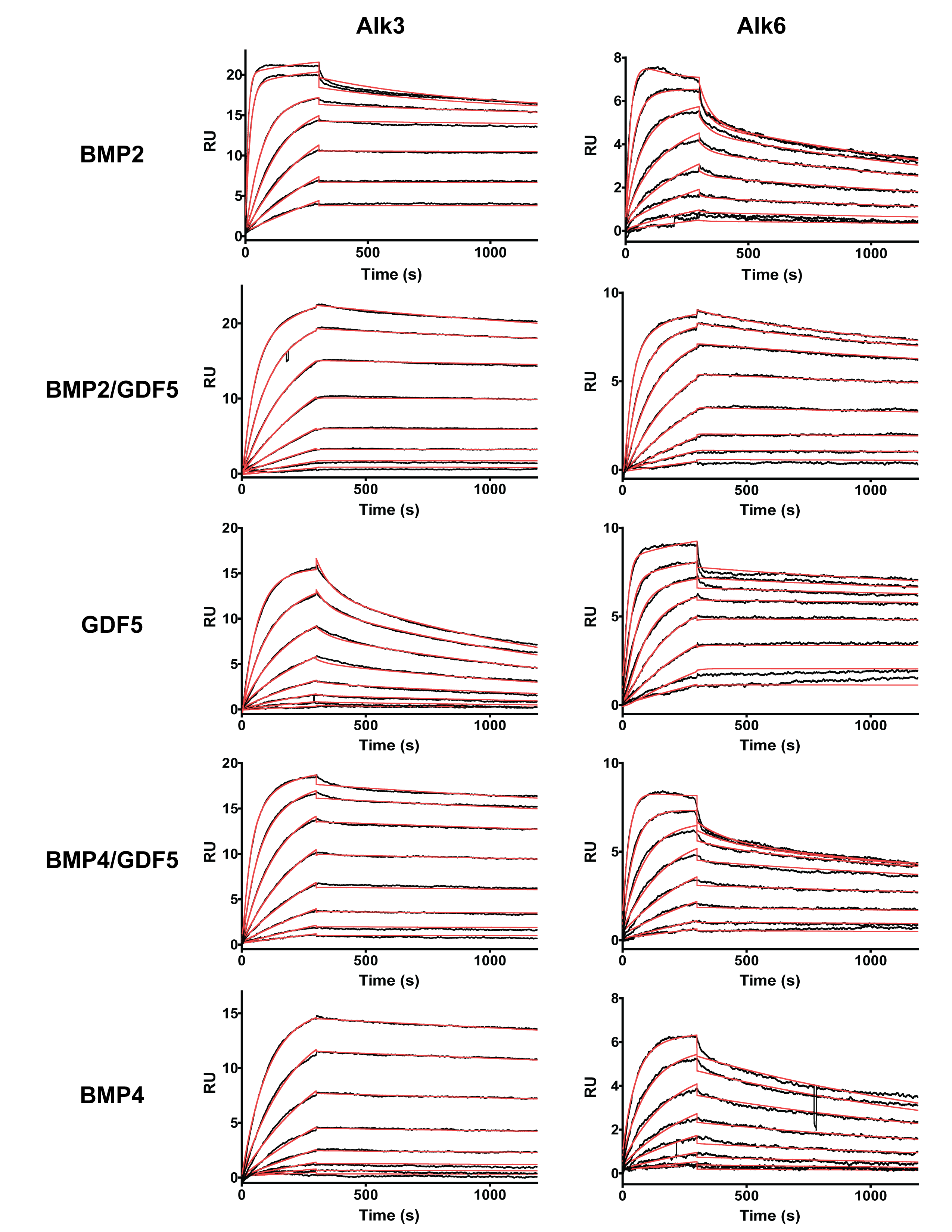

Supplement: Supplementary file 2 — Additional file 2: Figure S2. Representative SPR kinetic binding curves for ligand interactions with type 1 receptors. Representative plots of SPR binding curves of homodimeric and heterodimeric ligands to type 1 receptor Fc chimeras. Experimental traces (black) were fit using a 1:1 binding model and the fits are represented as a red line. All experiments were performed with variable ligand concentrations between 6.25 nM – 0.045 nM. [file 12915_2023_1522_MOESM2_ESM.tif]

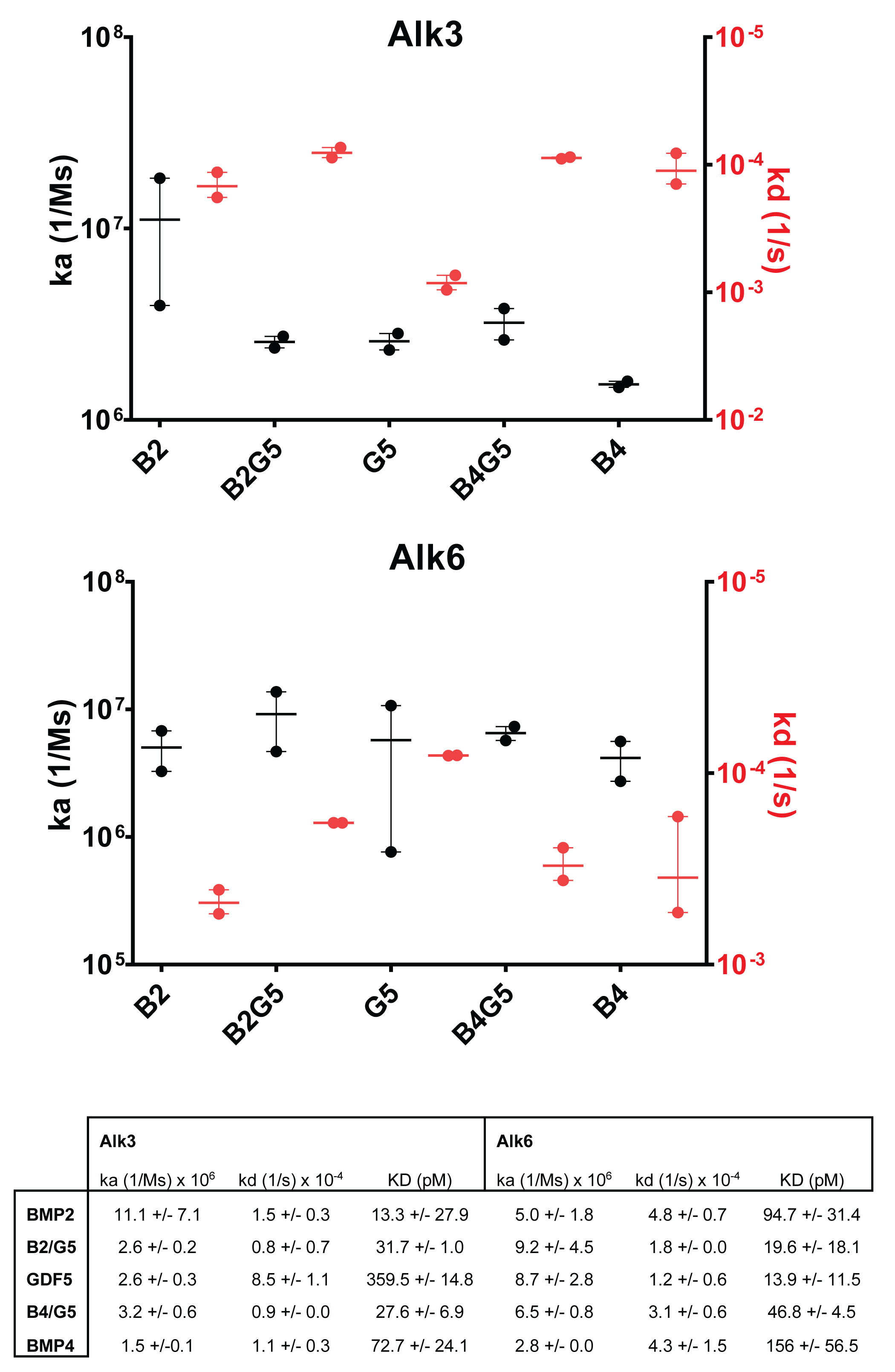

Supplement: Supplementary file 3 — Additional file 3: Figure S3. Calculated association and dissociation binding constants for ligand interactions with type 1 receptors, determined by kinetic SPR. Average of N=2 experiments. [file 12915_2023_1522_MOESM3_ESM.tif]

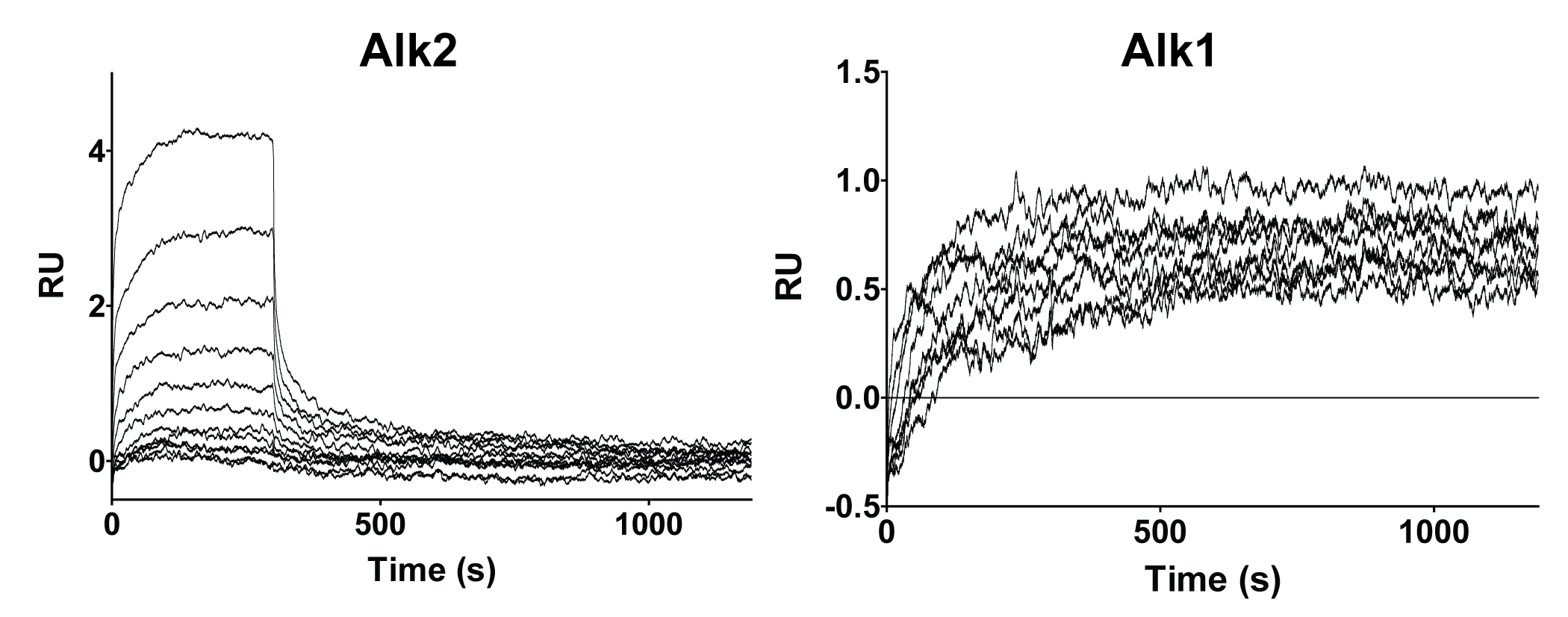

Supplement: Supplementary file 4 — Additional file 4: Figure S4. Representative SPR kinetic binding curves for ligand interactions with more type 1 receptors. Representative binding curves of BMP2 binding to Alk2 and Alk1, measured by SPR. All tested growth factors bound similarly to these receptors. All experiments were performed with variable ligand concentrations between 6.25 nM – 0.045 nM. [file 12915_2023_1522_MOESM4_ESM.tif]

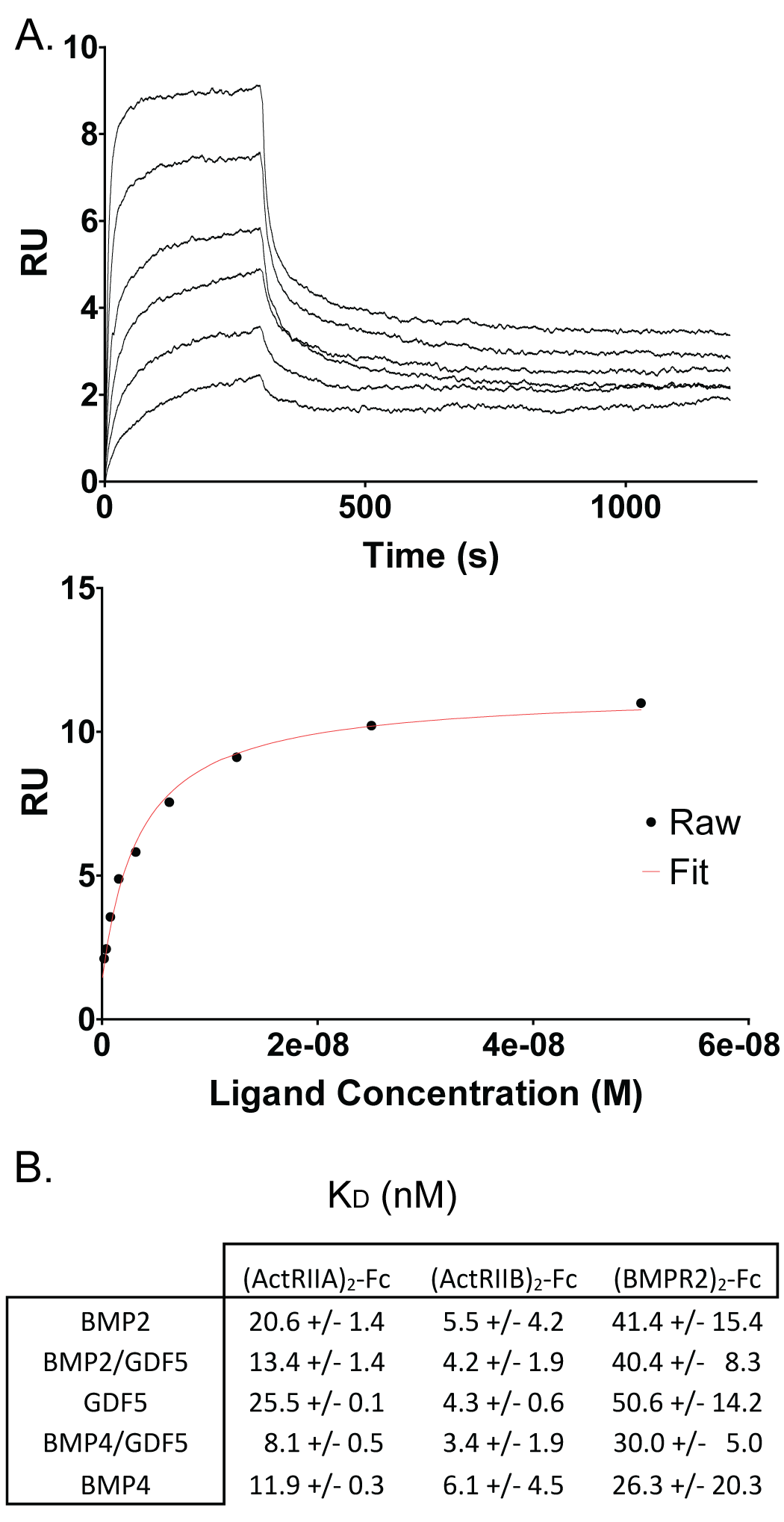

Supplement: Supplementary file 5 — Additional file 5: Figure S5. Steady-state analysis for ligand interactions with type 2 receptors. A) Representative T2 binding curve, GDF5 and (ActRIIB)2-Fc, and Steady-State binding fit. B) Table of binding constants (KD, in nM) of homodimeric and heterodimeric growth factors to type 2 receptors, determined by steady state analysis. Average of N=2 experiments. All experiments were performed with variable ligand concentrations between 100 nM – 0.195 nM (for ActRIIA and BMPR2) or 25 nM – 0.195 nM (ActRIIB). [file 12915_2023_1522_MOESM5_ESM.tif]

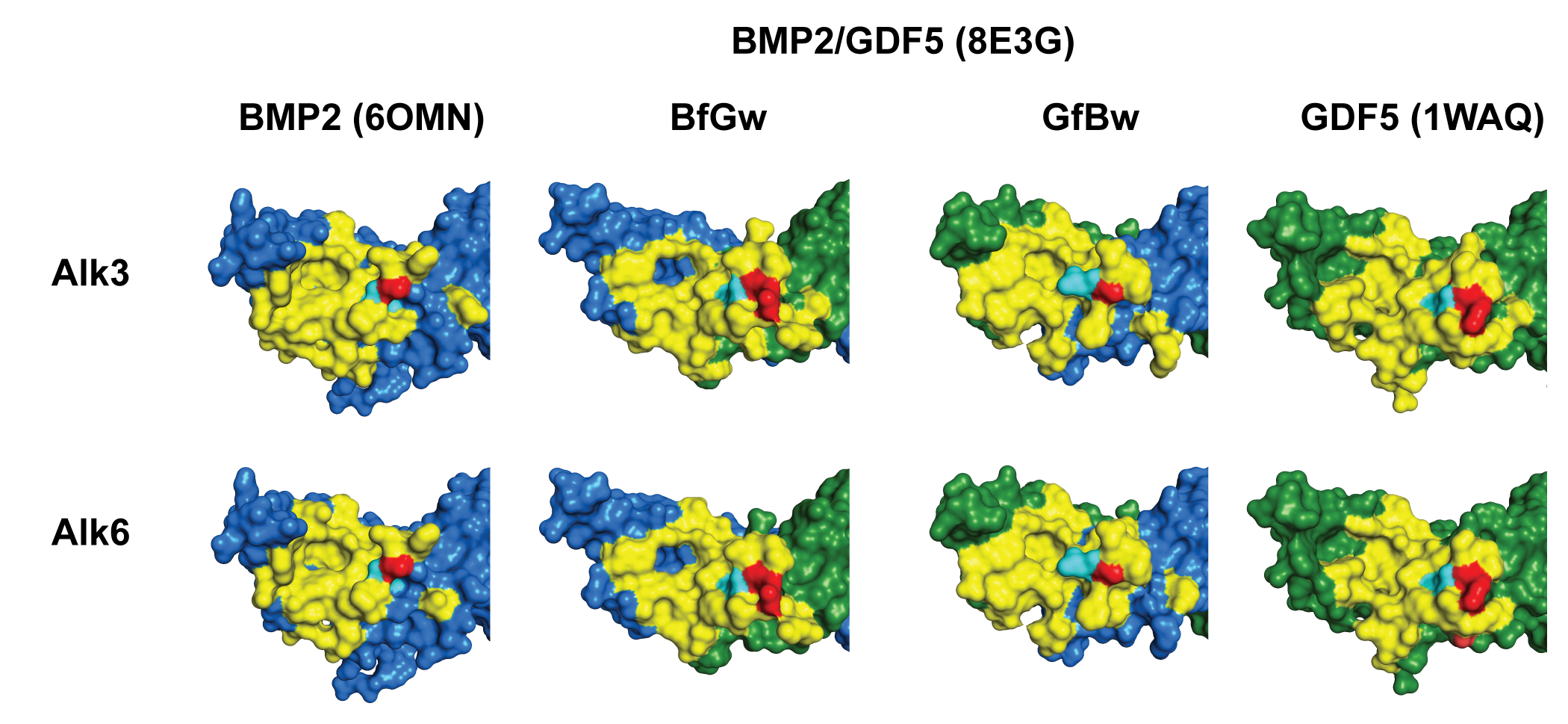

Supplement: Supplementary file 7 — Additional file 7: Figure S6. Comparison of type 1 receptor binding pockets of BMP2, GDF5, and BMP2/GDF5 heterodimer. The Alk3 and Alk6 extracellular domains from binary complex structures 1REW and 3EVS, respectively, were aligned (using PyMol) to homodomeric BMP2 (6OMN), GDF5 (1WAQ) or BMP2/GDF5 heterodimer (8E3G), based on the alignment of the ligands targeting the wrist helix of binding pocket [46, 63, 92]. Residues within 5Å of receptors colored yellow. Leucine required for type 1 binding (L333 in BMP2, L451 in GDF5) in cyan [67]. Residue implicated in Alk3 vs Alk6 binding preference in GDF5 in red (A334 in BMP2, R452 in GDF5) [26]. [file 12915_2023_1522_MOESM7_ESM.tif]

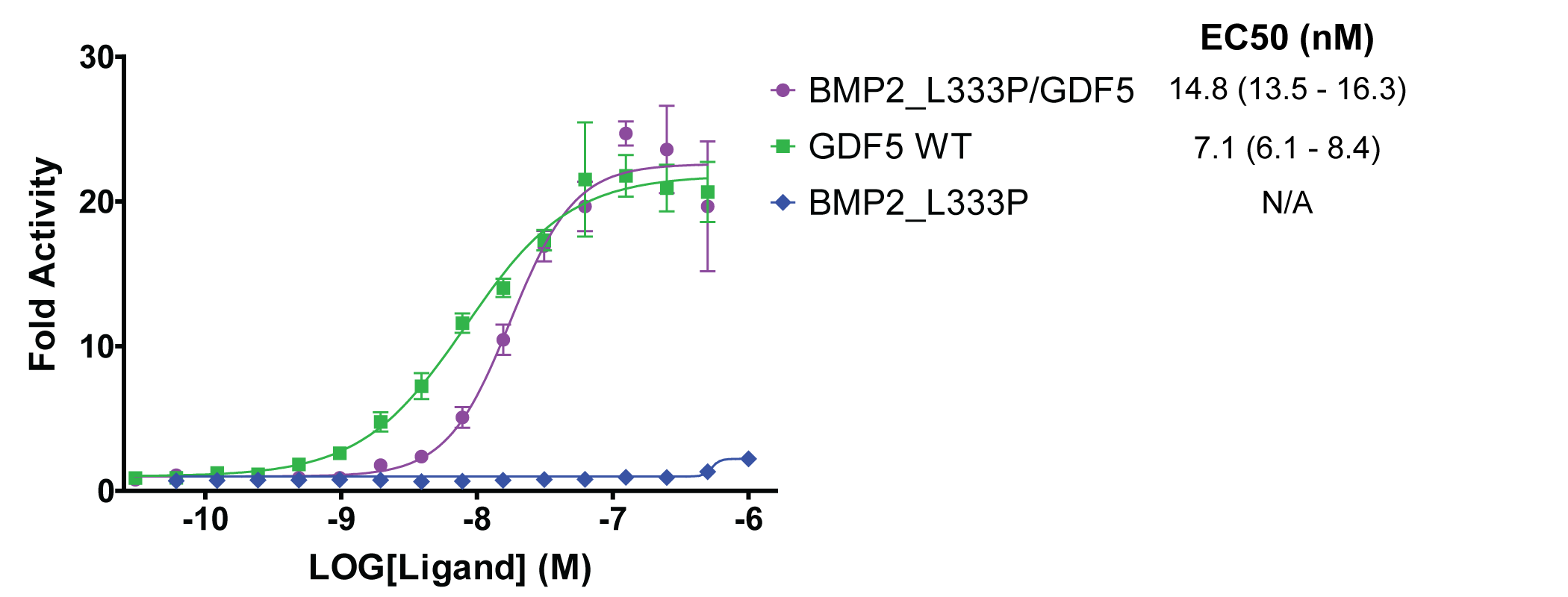

Supplement: Supplementary file 8 — Additional file 8: Figure S7. Luciferase reporter assay for mutant heterodimer. Luciferase Reporter Assay used to compare function of BMP2/GDF5 heterodimer (purple) to BMP2 homodimer (blue), GDF5 homodimer (green), or a combination of BMP2 and GDF5 homodimers (gray). Representative curves shown. Data normalized to untreated control and analyzed using GraphPad Prism using non-linear regression to determine EC50. Data tables display an average of N=3 experiments, with 95 % confidence range reported. [file 12915_2023_1522_MOESM8_ESM.tif]

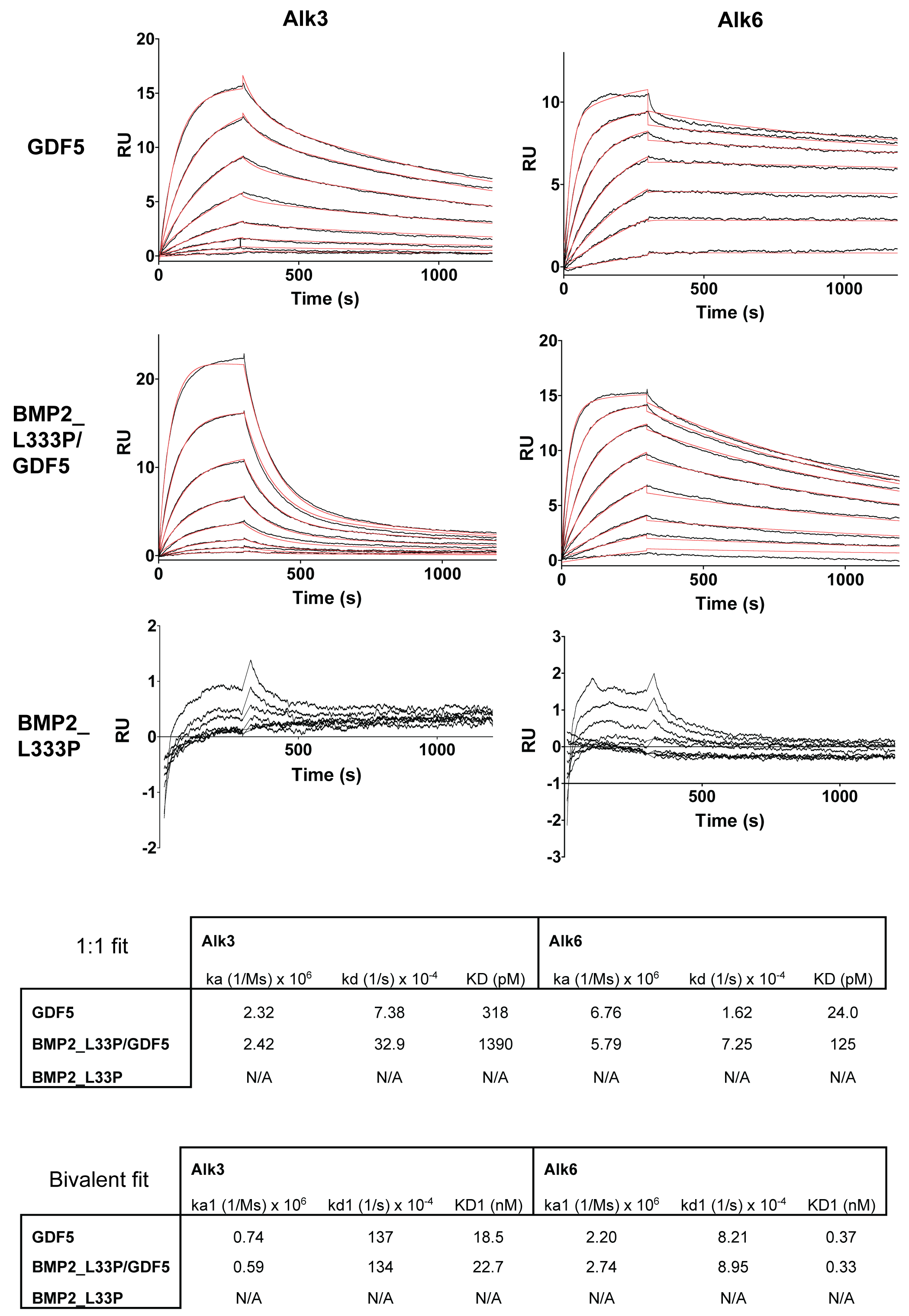

Supplement: Supplementary file 9 — Additional file 9: Figure S8. SPR kinetic binding curves for mutant heterodimer. SPR binding results for GDF5, BMP2_L333P, and BMP2_L333P/GDF5 heterodimer, analyzed for kinetic binding using both a 1:1 binding model and a Bivalent binding model. All experiments were performed with variable ligand concentrations between 6.25 nM – 0.045 nM. [file 12915_2023_1522_MOESM9_ESM.tif]
