# Supplementary material for: Formation and characterization of BMP2/GDF5 and BMP4/GDF5 heterodimers
Source: BMC Biol. 2023 Feb 1;21:16. doi: 10.1186/s12915-023-01522-4 (PMC9893541; doi:10.1186/s12915-023-01522-4)
Supplement: Supplementary file 6 — Additional file 6: Table S1. X-ray diffraction data and refinement statistics. [file 12915_2023_1522_MOESM6_ESM.docx]

| **BMP2/GDF5 heterodimer** | |  |
| --- | --- | --- |
|  |  |  |
| PDB Deposition Code: | 8E3G |  |
| **Data Collection** |  |  |
| Space Group | P 3_2_ 2 1 |  |
| Unit cell dimensions |  |  |
| ***a****,* ***b****,* ***c*** (Å) | 98.3, 98.3, 174.8 |  |
| a, b, g (°) | 90, 90, 120 |  |
| Wavelength (Å) | 1.0332 |  |
| Resolution (Å)^a^ | 76.6-2.8 (2.9-2.8) |  |
| R_merge_^a^ | 0.173 (1.363) |  |
| R_pim_^a^ | 0.055 (0.426) |  |
| Mn (I/sd)^a^ | 11.2 (2.3) |  |
| CC_1/2_ ^a^ | 0.995 (0.709) |  |
| Completeness (%)^a^ | 100 (100) |  |
| Redundancy^a^ | 11.0 (11.2) |  |
| Twin Fraction | 0.028 |  |
| Twin Law | h+k, -k, -l |  |
|  |  |  |
| **Refinement** |  |  |
| Resolution | 76.6-2.8 |  |
| No. reflections^a^ | 24,734 (2,428) |  |
| R_work_ | 0.193 |  |
| R_free_^b^ | 0.234 |  |
| Number of: |  |  |
| Non-hydrogen atoms | 3326 |  |
| Protein residues | 418 |  |
| Non-protein atoms | 69 |  |
| RMS Deviations^c^ |  |  |
| Bond lengths (Å) | 0.015 |  |
| Bond angles (°) | 1.336 |  |
| Ramanchandran^c^ |  |  |
| Favored (%) | 92.44 |  |
| Allowed (%) | 6.83 |  |
| Outliers (%) | 0.73 |  |
| Rotomer outliers (%)^c^ | 5.03 |  |
| Clashscore | 3.69 |  |
|  |  |  |
| B-factor (avg Å) | 62.68 |  |
|  |  |  |
| ^a^ Values in parentheses are for highest resolution shell | |  |
| ^b^ R_free_ calculated from 5% of initial total number of reflections | |  |
| ^c^ Determined by MolProbity | |  |
